# Microvesicle-associated and circulating microRNAs in diabetic dyslipidemia: miR-218, miR-132, miR-143, and miR-21, miR-122, miR-155 have biomarker potential

**DOI:** 10.1186/s12933-023-01988-0

**Published:** 2023-09-25

**Authors:** Miruna Nemecz, Diana Simona Stefan, Ioana Karla Comarița, Alina Constantin, Gabriela Tanko, Cristian Guja, Adriana Georgescu

**Affiliations:** 1 Institute of Cellular Biology and Pathology ‘Nicolae Simionescu’ of the Romanian Academy, Bucharest, Romania; 2 National Institute of Diabetes, Nutrition and Metabolic Disease ‘Prof. Dr. Nicolae Constantin Paulescu’, Bucharest, Romania

**Keywords:** Diabetic dyslipidemia, Microvesicles, microRNAs

## Abstract

**Background:**

Circulating MicroRNAs (miRNAs) carried by microvesicles (MVs) have various physiological and pathological functions by post-transcriptional regulation of gene expression being considered markers for many diseases including diabetes and dyslipidemia. We aimed to identify new common miRNAs both in MVs and plasma that could be predictive biomarkers for diabetic dyslipidemia evolution.

**Methods:**

For this purpose, plasma from 63 participants in the study (17 type 2 diabetic patients, 17 patients with type 2 diabetes and dyslipidemia, 14 patients with dyslipidemia alone and 15 clinically healthy persons without diabetes or dyslipidemia) was used for the analysis of circulating cytokines, MVs, miRNAs and MV-associated miRNAs.

**Results:**

The results uncovered three miRNAs, miR-218, miR-132 and miR-143, whose expression was found to be significantly up-regulated in both circulating MVs and plasma from diabetic patients with dyslipidemia. These miRNAs showed significant correlations with important plasma markers, representative of this pathology. Thus, MV/plasma miR-218 was negatively correlated with the levels of erythrocyte MVs, plasma miR-132 was positively connected with MV miR-132 and negatively with uric acid and erythrocyte plasma levels, and plasma miR-143 was negatively related with creatinine levels and diastolic blood pressure. Also, three miRNAs common to MV and plasma, namely miR-21, miR-122, and miR-155, were identified to be down-regulated and up-regulated, respectively, in diabetic dyslipidemia. In addition, MV miR-21 was positively linked with cholesterol plasma levels and plasma miR-21 with TNFα plasma levels, MV miR-122 was negatively correlated with LDL-c levels and plasma miR-122 with creatinine and diastolic blood pressure and positively with MV miR-126 levels, MV miR-155 was positively associated with cholesterol and total MV levels and negatively with HDL-c levels, whereas plasma miR-155 was positively correlated with Il-1β plasma levels and total MV levels and negatively with MV miR-223 levels.

**Conclusions:**

In conclusion, miR-218, miR-132, miR-143, and miR-21, miR-122, miR-155 show potential as biomarkers for diabetic dyslipidemia, but there is a need for more in-depth studies. These findings bring new information regarding the molecular biomarkers specific to diabetic dyslipidemia and could have important implications for the treatment of patients affected by this pathology.

## Background

One of many reasons for the severity of vascular disease in diabetic patients is dyslipidemia, also known as diabetic dyslipidemia (DDLP) and defined as lipoprotein metabolism disorder characterized by elevated total cholesterol, the “bad” low-density lipoprotein (LDL) cholesterol and the triglyceride concentrations, and a decrease in the “good” high-density lipoprotein (HDL) cholesterol levels in the blood [1]. In diabetes, both small and large vessels are affected by lipid imbalance. In type 2 diabetes (T_2_D), characterized by impaired insulin sensitivity, the lipid phenotype includes hypertriglyceridemia, reduced plasma HDL cholesterol and the conversion of LDL to a more atherogenic lipoprotein, termed small dense LDL [2]. However, the dominant lipid defect in insulin resistance remains hypertriglyceridemia, a consequence of increased production and decreased release of triglyceride-rich lipoproteins in fasting as well as in non-fasting state [3]. This feature is commonly associated with a reduction in HDL and an increase in small dense LDL levels [4]. Conventionally low HDL levels were regarded as a consequence of insulin resistance and diabetes. Other studies indicated that low HDL may result in or exacerbate abnormal glucose homeostasis [5]. Even though impaired insulin sensitivity is considered to be the main cause for diabetic dyslipidemia, hyperglycemia alone cannot entirely justify the lipid changes, often detected many years before clinical diagnosis of prediabetes in individuals with normal glucose concentrations [6]. Therefore, unlike type 1 diabetes, lipid metabolism dysregulation characteristic for T_2_D cannot become completely corrected with glycemic control.

In addition to the known specific biochemical profile of diabetic or dyslipidemic patients, some of the most promising biomarkers could be extracellular vesicles (EVs), particularly microparticles (MPs) or microvesicles (MVs), and microRNAs (miRNAs).

MVs are larger EVs, which together with exosomes (smaller EVs) have important roles within our body. All EVs are quite similar, however, their different way of emerging is very important for their function. MVs, are vesicles of about 100 to 1000 nm in diameter, released by most of the activated or apoptotic cells, found both in tissues and body fluids and involved in cell-to-cell communication. Compared to exosomes, MVs do not require exocytosis for their formation and release. They are generated by the outward budding of a plasma membrane domain, as a result of the increase in intracellular Ca^2+^ concentration leading to phosphatidylserine (PS) exposure. Therefore, their cargo originates from the cytosolic face of the cell, where many proteins and RNAs involved in cell-to-cell interaction can be found [7–10]. The specific molecular signature of MVs consists of PS as a key identifier, several surface proteins which are specific to paternal cells and RNA, including miRNAs [11]. MVs express numerous features of cells of origin including specific surface antigens and receptors, so they were named according to the cells they come from, for example endothelial, platelet and leukocyte MVs [12]. In vitro studies confirmed that, high glucose concentration activates endothelial cells (ECs) to discharge significant amount of MVs, called endothelial microvesicles (EMVs), with a distinct molecular composition [13, 14]. These endothelial membrane-derived vesicles are also reported to carry regulating molecules crucial in cell-to-cell communication. All types of MVs are endowed with the ability to transport a specific class of miRNAs in circulation and transfer them to certain cells, such as ECs, endothelial progenitor cells (EPCs), through a tightly regulated process [15]. Following, the miRNAs transferred by MVs could play an important role in the regulation of biological processes involved in the vascular complications in diabetes, turning MVs in key vesicles of the processes underlying intercellular communication in different pathologies including diabetic dyslipidemia [16]. The miRNAs are known as small non-coding RNAs which regulate gene expression post-transcriptionally through the inhibition of translation and/or the degradation of messenger RNA (mRNA) and they have been found in several body fluids, including blood, circulating EVs or associated with lipoproteins or protein complexes [17, 18]. The levels of miRNAs are modified during disease progression and miRNAs’ circulating profiles often reflect their modified expression in the tissue of origin or indicate increased intercellular communication [19]. There are clear data on a possible use of miRNAs as diagnostic biomarkers and therapeutic targets for the endothelial dysfunction in hypertensive patients [20]. In diabetes, alterations of miRNA expressions were assumed that could disturb the production of insulin and occurrence of diabetic complications [21]. In addition, differences in the abundance of pro- and anti-angiogenic miRNAs in the MV-enriched plasma fraction were reported in patients with T_2_D, suggesting that development of vascular complications is due to impaired angiogenesis in such patients [22]. Reportedly, miRNAs associated with MVs regulate cellular mechanisms involved in vascular homeostasis in the progression of diabetic dyslipidemia [7, 23–25]. The most recent methods of diagnosis and therapy target EV and miRNA technologies. We hypothesized that detection of the changes in plasma MV components could be used as early biomarkers to prevent the irreversible effects of diabetic dyslipidemia. The aim of this study was to search whether the newly discovered modifications of the circulating MVs and plasma miRNAs could be predictive for diabetic dyslipidemia evolution. However, to date, MV-associated and circulating miRNAs are not applied as biomarkers in clinical settings for diabetic dyslipidemia.

## Methods

### Patients and control subjects

A total number of 63 male and female participants, including healthy volunteers, patients with dyslipidemia and patients with type 2 diabetes, selected from National Institute for Diabetes, Nutrition and Metabolic Disease ‘Prof. Dr. Nicolae Constantin Paulescu’, Bucharest, Romania, and who signed the consent form, were enrolled in the study and divided into 4 experimental groups.

Diabetic patients of similar age and body mass index (BMI) and different degrees of cholesterol levels were divided into 2 experimental groups: (1) patients with type 2 diabetes (T_2_D group, n = 17) and (2) patients with type 2 diabetes and dyslipidemia (T_2_D-DLP group, n = 17). As control, two experimental groups of participants were also included: (3) patients with dyslipidemia alone (DLP group, n = 14, control for T_2_D-DLP group) and (4) clinically healthy persons without diabetes or dyslipidemia (C group, n = 15, control for DLP, T_2_D and T_2_D-DLP groups).

The division of patients into diabetics and non-diabetics was made according to American Diabetes Association (ADA) criteria by measuring fasting plasma glucose (FPG), a test done first thing in the morning, before breakfast, after a minim of 8 h fasting (means not eating and drinking anything, except water, for a period of 8 h). Diabetes was diagnosed at fasting blood glucose of greater than or equal to 126 mg/dL, and normal was considered at fasting blood glucose under 100 mg/dL.

### Clinical and metabolic measurements

The blood was collected by venipuncture from each volunteer or patient included in the study and clinical characteristics (gender, age, BMI, systolic/diastolic blood pressure, heart rate and several biochemical data: transaminases, urea, creatinine, uric acid, hemoglobin and leucocyte) were carried out**.** Also, to state the metabolic profile, consisting of plasma glucose, glycated hemoglobin (HbA1C), total cholesterol, LDL-cholesterol, HDL-cholesterol, triglyceride, was determined. All tests were performed on sera using Cobas Integra 400 plus analyzer (Roche Diagnostics). Additional blood samples were collected from every participant in the study and stored at − 70 °C for further investigations.

### Enzyme-linked immunosorbent assay

Using specific Elisa kits (R&D Systems), the circulating levels of inflammatory factors, interleukin 6 (IL-6), interleukin 1beta (IL-1β), interferon gamma (IFNγ), tumor necrosis factor alfa (TNFα), were measured in sera as indicated by the manufacturer. Elisa plates were analyzed on spectrophotometer (Tecan Infinite M200 PRO).

### Microvesicle isolation

Peripheral blood have been collected on anticoagulant (2 vacutainer tubes, blood collection tubes with EDTA K3) and total circulating MVs were separated from plasma by sequential centrifugations, according to the protocol optimized after the one described by [9], as follows: the peripheral blood was centrifuged at 2500*g* for 10 min at 4 ºC to collect the platelet poor plasma (PPP) from supernatant; subsequently, 200 µL PPP were centrifuged at 16000*g* for 5 min at 4 ºC to remove residual platelets, apoptotic bodies and collect the platelet free plasma (PFP) in supernatant; finally, MVs obtained in the pellet by centrifugation of PFP at 20000*g* for 90 min at 4 ºC were washed twice (20000 g, 90 min, 4 ºC) with phosphate buffered saline (PBS), re-suspended in PBS (~ 100 μL) and stored at − 80 °C until further analysis [19].

### Microvesicle characterization

Zetasizer Nano ZS (Malvern Instruments, Malvern, United Kingdom) equipped with a solid-state He–Ne laser at 633 nm wavelength, was used to establish the size distribution for MVs. The measurements were carried out in triplicates on MV suspension (10 μL) diluted to 1 mL of PBS. Zetasizer software version 7.03 was used in order to process and analyze the obtained data.

The fluorescence and electron microscopy were applied to investigate the structure and size of MVs. For fluorescence microscopy analysis, MVs were stained with red fluorescent membrane linker dye PKH26 (Invitrogen, Waltham, MA, United States) according to the manufacturer’s instructions and the protocol described by [26]. In brief, MV suspension (20 μL) in PBS, mixed with diluent C (1 mL) and PKH26 (1 mL) stock solution (4 × 10^−6^ M/L) was incubated at 37 °C for 5 min, the reaction was stopped by adding an equal volume of fetal bovine serum (FBS). MVs were analyzed under a Zeiss Axiovert microscope (Carl Zeiss, Jena, Germany). For electron microscopy analysis, MVs were subjected to the negative staining technique consisting of incubating 5 μL of MVs in suspension for 2 min on carbon-coated copper grids (100 mesh, Agar Scientific) and their subsequent staining with 2% uranyl acetate at room temperature (RT). Image acquisition was carried out at 18˚C using a Talos F200C Transmission Electron Microscope (Fei/Thermo Fisher Scientific Talos F200C TEM) [27].

### Microvesicle quantification and analysis

A Gallios Flow Cytometer (Beckman Coulter Life Sciences, CA, USA) was used to quantify the levels of MVs in circulation and analyze them according to the cells of origin from which they come.

To quantify MVs, 10 µL of MVs re-suspended in PBS were mixed with 10 µL of counting beads (1000 beads/µL, 10 µm diameter), incubated for 40 min at RT in the dark, and 100 µL of PBS were added at the time of reading to the flow cytometer for 60 s. The number of MVs (0.1–1 µm diameter) was calculated using the formula: MVs as events/µL = [(MV count/bead count) × bead concentration/µL] × MV purity/100, where: MV count and bead count were collected from the dot-plot representations (X = forward-scatter intensity, Y = side-scatter intensity), and MV purity was evaluated as follows: 10 µL of re-suspended MVs were incubated with 2.5 µL of Annexin V-FITC antibody (binds PS on MVs) in the presence of 2 mM CaCl2 for 40 min at RT in the dark, diluted with 100µL of PBS and read for 60 s at flow cytometer [15, 19].

To analyze MVs according to the types of cells from which they originate in the circulation, fluorescent antibodies (all from SantaCruz Biotechnology, Inc.) against specific cell surface markers were used: CD41 (Integrin alpha-IIb)—specific marker for platelets; CD144 (VE-cadherin)—specific marker for endothelial cells; CD14—specific marker for monocytes; CD18 (Integrin beta chain-2)-specific marker for leucocytes; CD235a (glycophorin A)-specific marker for erythrocytes. In brief, 10 µL of re-suspended MVs were incubated with 2.5 µL of Annexin V-FITC antibody and specific surface marker fluorescent antibody for 40 min at RT in the dark, diluted with 100 µL of PBS and analyzed with a 5000-event cutoff. Data were analyzed with Kaluza Flow Cytometry Analysis Software v2.1 (Beckman Coulter Life Sciences, Indianapolis, IN, USA) and expressed as a percentage of MVs double labeled with Annexin V and antibody specific to the cell of origin. The isotype control experiments were performed as well.

### RNA isolation and quantification

Total RNAs, including miRNA, were isolated from 200 µL plasma or purified MVs (obtained from 400 µL plasma and re-suspended in 100 µL PBS) using miRNeasy Serum/Plasma Kit (Qiagen, Hilden, Germany), according to the manufacturer’s instructions. After, RNAs were eluted in 14 μL RNase-free water. The RNA purity and concentration were evaluated by spectrophotometry using NanoDrop 2000c (Thermo Fisher) and isolated RNAs were kept at − 80 °C until further analysis.

### Analysis of miRNA expression in plasma and circulating MVs

TaqMan technology was used to measure the levels of hsa-miR-21-5p (ID: 000397), hsa-miR-34a-5p (ID: 000426), hsa-miR-122-5p (ID: 002245), hsa-miR-126-3p (ID: 002228), hsa-miR-132-3p (ID: 000457), hsa-miR-143-3p (ID: 002249), hsa-miR-155-5p (ID: 002623), hsa-miR-200b-3p (ID: 002251), hsa-miR-212-5p (ID: 000515), hsa-miR-218-5p (ID: 000521) and hsa-miR-223-3p (ID: 002295) (all from Qiagen, Hilden, Germany) in plasma and circulating MVs.

For this purpose, 50 ng RNA were consumed to generate single stranded cDNA using specific stem-loop primers of TaqMan miRNA Assays (RT primer-for transcription) and TaqMan MicroRNA Reverse Transcription Kit. cDNA was amplified with TaqMan Gene expression Master Mix and specific stem-loop primers of TaqMan miRNA Assays (TM primer-for amplification) to profile mature miRNAs by quantitative reverse transcription polymerase chain reaction (qRT-PCR). The investigated miRNA-specific RT/TM primers were used in separate reactions performed in duplicate. For the quantification of miRNAs in plasma it was used synthetic cel-miR-39-5p (ID 000200) (Qiagen, Hilden, Germany) as an exogenous control. This was added as spike-in during RNA isolation from plasma as previously described [28, 29]. Regarding miRNA quantification in circulating MVs, U6 small nuclear RNA (U6 snRNA) (ID001973) (Qiagen, Hilden, Germany) was used as an endogenous control and it was added in separate reactions in the same way as all the other miRNA-specific RT/TM primers were added.

All reactions were performed using Veriti real-time PCR system (for Reverse Transcription Assay) and VIIA7 real-time PCR system (for qRT-PCR Assay), both from Applied Biosystems (Life Technologies Foster City, CA, USA). The data were analyzed using VIIA7 Software v1.2 (Applied Biosystems) with the automatic quantification cycle (Cq) settings. Raw data passed all quality control checks and were filtered by applying cut-offs of Cq < 35 (measure of a good amplification).

MiRNA levels were calculated using the 2^−ΔCq^ method and multiplied by 10^3^ (for plasma). The expression level of each miRNA of interest was normalized to exogenous cel-miR-39-5p for plasma or endogenous snRNU6 for MVs, as previously re-ported [9, 19].

### Statistical analysis

Statistical analysis was performed using GraphPad Prism 7.02 software (GraphPad Software Inc., San Diego, CA, USA). The results were expressed as the means ± standard deviation (SD). Differences between experimental groups were determined by one-way ANOVA followed by Bonferroni multiple comparison tests. Statistical significance was set at p < 0.05 (^&^, *, ^#^), p < 0.01 (^&&^, **, ^##^) and p < 0.001(^&&&^, ***, ^###^). Spearman rank correlation test was used to assess relationships among continuous variables. A p value less than 0.05 (typically ≤ 0.05) was considered statistically significant.

## Results and discussion

### The metabolic and inflammatory profile of diabetic patients with dyslipidemia

#### Demographic and clinical characteristics of the patients

The patients included in the study were divided according to their clinical characteristics into the following groups: (1) patients with type 2 diabetes (T_2_D group); (2) patients with type 2 diabetes and dyslipidemia (T_2_D-DLP group); (3) patients with dyslipidemia alone (DLP group), and (4) clinically healthy persons without diabetes or dyslipidemia (C group).

As shown in Table 1, both T_2_D patients and T_2_D-DLP patients had poor glycemic control as revealed by significant higher fasting plasma glucose and HbA1c values compared with healthy individuals (C group). Also, patients with dyslipidemia (DLP group) and patients with diabetic dyslipidemia (T_2_D-DLP group) exhibited significantly higher levels of triglycerides compared with control group. The rest of the metabolic parameters did not significantly differ among the 4 experimental groups. Both systolic and diastolic blood pressure were significantly higher in DLP patients, while T_2_D patients had only systolic blood pressure significantly higher, compared to healthy subjects. Diabetic patients with dyslipidemia (T_2_D-DLP group) did not show significant changes in systolic and diastolic blood pressure values (Table 1).

**Table 1:** Clinical characteristics of different groups of patients included in the study

	C (n = 15)	DLP (n = 14)	T2D (n = 17)	T2D-DLP (n = 17)
Anthropometric data				
Gender (F/M)	3F/2 M	3F/1 M	2F/5 M	8F/9 M
Age (years)	43 ± 8	60 ± 11	64 1 ± 13	58 ± 9
Onset of disease (years)	0	0	1 → 10	1 → 14
BMI (kg/m^2^)	29 ± 7	35 ± 2	29 ± 4	32 ± 5
Metabolic waste products				
Serum urea (mg/dL)	24 ± 6	32 ± 8	35 ± 14	33 ± 10
Serum creatinine (mg/dL)	1 ± 0	1 ± 0	1 ± 0	1 ± 0
Serum uric acid (mg/dL)	5 ± 2	7 ± 2	5 ± 2	5 ± 1
Serum hemoglobin (g/dL)	14 ± 1	15 ± 1	15 ± 1	14 ± 1
Erythrocytes (mm^3^)	5,040,000 ± 183,847	5,210,000 ± 226,274	5,152,000 ± 388,361	4,913,333 ± 795,831
Platelets (mm^3^)	233,666 ± 68,233	244,375 ± 164,421	193,714 ± 52,392	253,316 ± 55,037
Leucocytes (mm^3^)	6496 ± 2409	10,452 ± 4060	7634 ± 2779	8399 ± 2302
Monocytes (mm^3^)	623 ± 68	713 ± 396	664 ± 75	755 ± 160
Metabolic parameters				
Fasting plasma glucose (mg/dL)	80 ± 10	88 ± 9	152 ± 48^a^	144 ± 28^a^
HbA1c (%)	5 ± 0.6	6 ± 0.8	7 ± 1.4^a^	7 ± 2ª
Serum triglycerides (mg/dL)	104 ± 27	212 ± 17^a^	125 ± 11	167 ± 11^a,b^
Serum cholesterol (mg/dL)	200 ± 32	204 ± 71	171 ± 39	162 ± 48
HDL cholesterol (mg/dL)	54 ± 19	38 ± 7	44 ± 14	40 ± 5
LDL cholesterol (mg/dL)	104 ± 17	127 ± 27	101 ± 35	129 ± 29
TGO (UI/L)	22 ± 10	20 ± 5	19 ± 18	22 ± 12
TGP (UI/L)	24 ± 13	23 ± 17	22 ± 13	26 ± 15
Heart function				
Systolic blood pressure (mmHg)	113 ± 10	152 ± 19^a^	132 ± 10ª	133 ± 15
Diastolic blood pressure (mmHg)	68 ± 10	96 ± 11^a^	81 ± 13	76 ± 11
Heart rate (bpm)	71 ± 9	93 ± 8	94 ± 11	84 ± 10

Data presented as mean ± SD (one-way ANOVA and Bonferonni)

C control, DLP dyslipidemia, T2D type 2 diabetes, T2D-DLP diabetic dyslipidemia

^a^p < 0.05 vs C

^b^p < 0.05 vs T2D

#### Plasma inflammatory markers of the patients

**Fig. 1:**
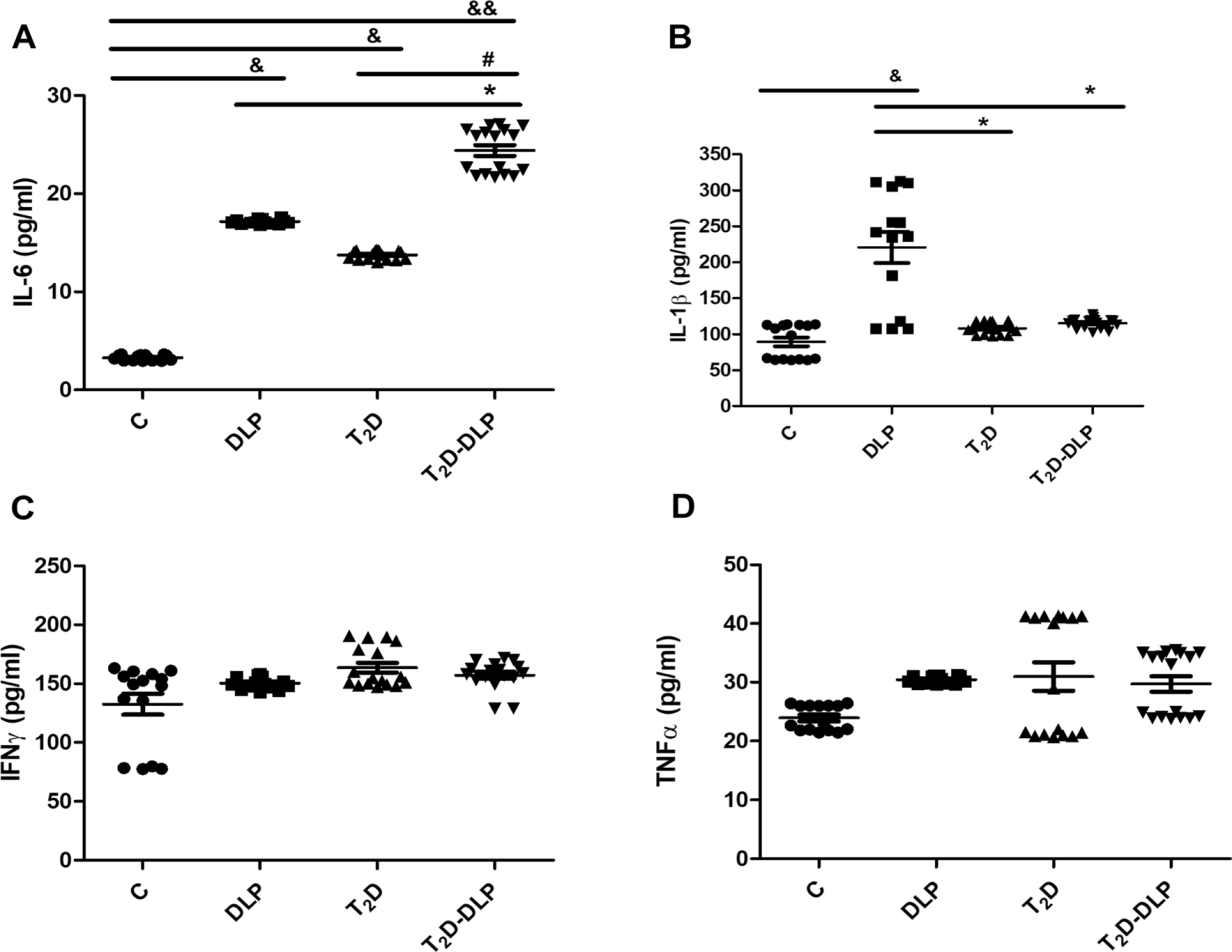
Pro-inflammatory levels of IL-6 (**A**), IL-1β (**B**), IFNγ (**C**) and TNF-α (**D**) in plasma from patient groups DLP(n = 14), T_2_D(n = 17), T_2_D-DLP(n = 17) and group C (n = 15) of healthy subjects, measured by Elisa method. Data are expressed by means ± SD. ^*&*^*p* < *0.05* (vs C), ^*&&*^*p* < *0.01* (vs C), *p < 0.05 (vs DLP), ^#^*p* < 0.05 (vs T_2_D) (One-Way ANOVA with Bonferroni's Multiple Comparison Test)

Elevated values of pro-inflammatory molecules correlated or not with reduction of anti-inflammatory ones, were usually associated with dyslipidemia as well as with diabetes. Also, combined elevation of inflammatory cytokines, led not only to a stronger inflammatory environment, but it could even be essential for the appearance of the diabetic condition. Interestingly, it was shown that patients with elevated levels of IL-6, IL-1β and TNFα had considerably increased risk of developing T_2_D compared with individuals with higher levels of IL-6 alone. Pro-inflammatory cytokine, IL-1β, has been described as a severe inducer of beta-cell damage. It has been shown that, long lasting elevated IL-1β levels in both obese and T_2_D patients cause beta-cell dysfunction [30, 31], while low levels of IL-1β lead to β-cell proliferation, beta-cell apoptosis inhibition and enhance glucose-stimulated insulin secretion [32]. However, i*n vitro* studies showed that IL-1β is essential not only for beta-cell compensation, but also for β-cell failure as well [32, 33], meaning that the IL-1β levels may vary in diabetes, depending on the disease stage. Also, age, BMI and various genetic and hormonal factors, can modify the levels of IL-1β in T_2_D [30, 34], and the treatment with metformin or acarbose decreases IL-1β protein levels in T_2_D patients [30, 35]. The levels of inflammatory cytokines, IL-6, IL-1β, IFNγ, TNFα, were measured in plasma of the individuals evaluated in our study (Fig. 1).

As shown in Figure, T_2_D patients had significantly elevated plasma levels of IL-6, while patients with dyslipidemia alone (DLP group) had significantly increased levels of both IL-6 and IL-1β, when these were compared with the levels measured in healthy individuals (C group). These results are in agreement with those reported by [36] that showed that an imbalance between IL-6 and IL-10 expression led to increased risk of developing T_2_D, but at the same time they are in disagreement with the results that showed that increased levels of IL-6 associated with those of IL-1β represent an increased risk for developing T_2_D [37]. In our study, T_2_D was associated only with an increase in plasma concentrations of IL-6 (Fig. 1A), not with increases in IL-1β, IFNγ and TNFα plasma levels (Fig. 1B–D). In addition, diabetic patients with dyslipidemia (T_2_D-DLP group) showed significant increases in plasma concentrations of IL-6 both compared to group C and compared to groups T_2_D and DLP, meaning that both diabetes and dyslipidemia had a cumulative effect on IL-6 plasma levels. (Fig. 1A). It should be noted that, the plasma concentrations of the other investigated inflammatory markers (IL-1β, IFNγ, TNFα) remained unchanged in diabetic dyslipidemia (T_2_D-DLP group), although IL-1β increased significantly in the patients who had only dyslipidemia (DLP group) (Fig. 1B–D). In addition, a positive correlation was observed between IL-1β levels and systolic blood pressure (r = 0.90; p = 0.03) in patients with diabetic dyslipidemia (Table 2).

**Table 2:** Significant correlations among circulating/plasma inflammatory cytokines and relevant clinical parameters in the diabetic dyslipidemia context

T_2_D-DLP group		Cytokine plasma levels			
		IFNγ	TNFα	IL1β	IL6
Clinical measurements ↓	n	17	17	17	17
TC	r	− 0.610	− 0.184	− 0.324	− 0.013
	P	0.264	0.763	0.593	0.974
HDL-c	r	0.531	0.461	0.162	− 0.312
	P	0.351	0.423	0.783	0.604
LDL-c	r	0.504	0.123	− 0.594	0.293
	P	0.390	0.843	0.297	0.624
TG	r	− 0.583	− 0.612	0.053	0.594
	P	0.293	0.261	0.924	0.292
TGO	r	− 0.452	− 0.581	0.082	0.711
	P	0.432	0.302	0.894	0.170
TGP	r	**0.821**	0.120	− 0.443	0.563
	P	**0.080**	0.843	0.453	0.322
Fasting Glucose	r	0.502	0.284	− 0.453	− 0.573
	P	0.383	0.643	0.434	0.314
HbA1c	r	0.573	− 0.411	0.413	0.073
	P	0.311	0.484	0.492	0.903
Hemoglobin	r	0.083	− 0.453	0.256	0.784
	P	0.894	0.433	0.673	0.103
Creatinine	r	− 0.024	− 0.132	− 0.253	0.754
	P	0.964	0.822	0.684	0.133
Urea	r	− 0.373	− 0.351	− 0.273	0.711
	P	0.532	0.553	0.652	0.174
Uric acid	r	− 0.674	− 0.684	0.253	0.201
	P	0.213	0.203	0.681	0.743
Erythrocytes	r	− 0.504	− 0.633	0.150	0.184
	P	0.392	0.252	0.804	0.773
Platelets	r	0.334	**0.811**	− 0.624	− 0.590
	P	0.581	**0.094**	0.250	0.293
Leucocytes	r	***0.881***	0.260	− 0.171	0.371
	P	***0.040***	0.664	0.770	0.532
Monocytes	r	− 0.752	0.093	0.314	− 0.773
	P	0.144	0.884	0.604	0.123
SBP (systolic blood pressure)	r	0.224	− 0.783	***0.900***	0.182
	P	0.713	0.113	***0.031***	0.750
DBP (diastolic blood pressure)	r	− 0.074	− 0.022	0.303	0.660
	P	0.893	0.961	0.624	0.211

Spearman rank correlation test was used to assess relationships among variables. Bolded coefficients show a powerful correlation, and of them only italic underlined coefficients are significant (p < 0.05)

### Elevated levels of circulating microvesicles in diabetic patients with dyslipidemia

#### Characterization, quantification and analysis of plasma microvesicles

**Fig. 2:**
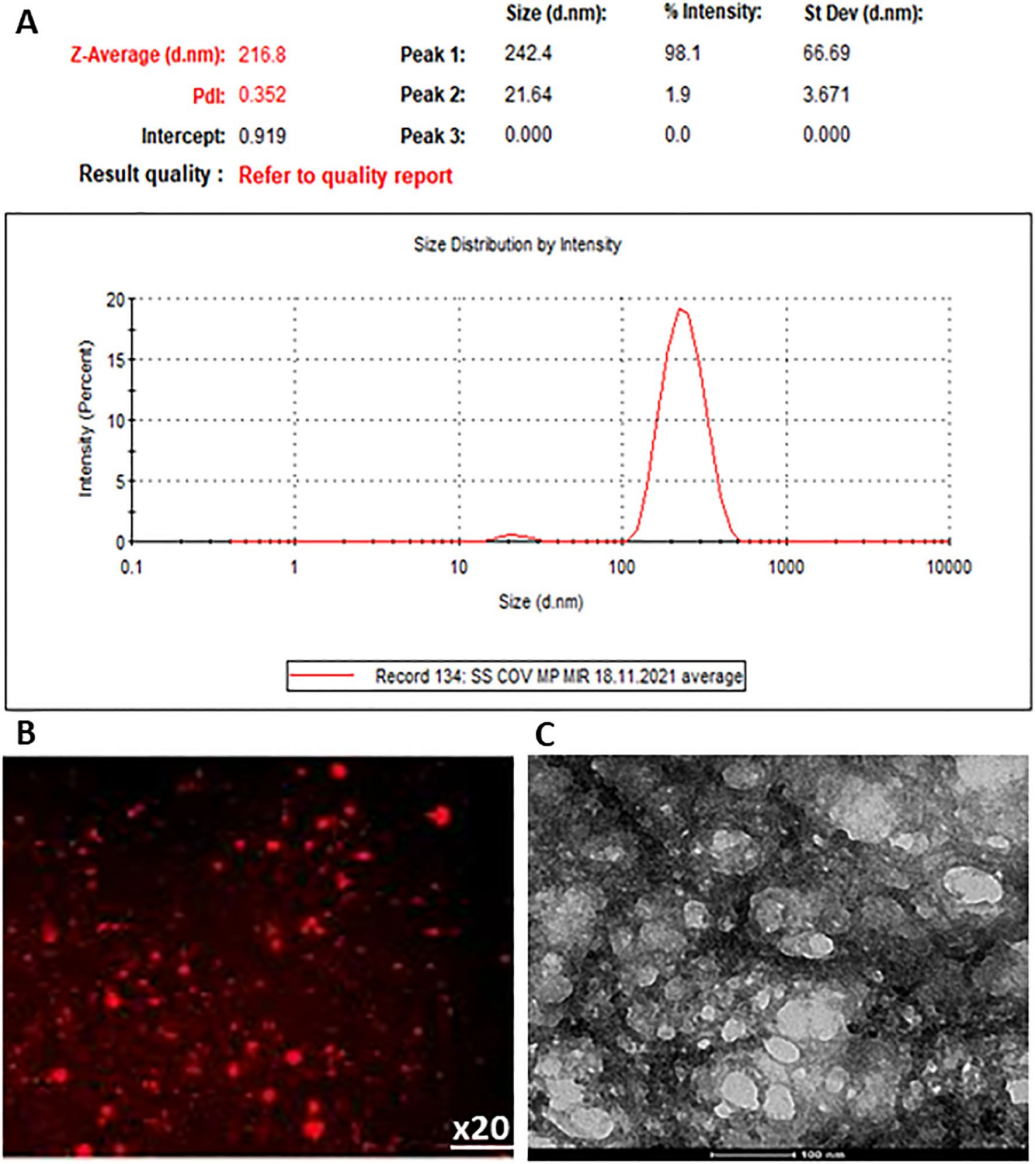
Analysis of plasma MVs by Zetasizer Nano ZS showing a large population represented by one peak at size range of ~ 100–1000 nm, specific to MVs (Y-axis represents the number of MVs and X-axis the size of MVs) (**A**). Examination of plasma MVs by fluorescence microscopy using PKH26 staining technique (**B**), and electron microscopy using negative staining technique (**C**)

MVs were isolated through successive centrifugations from plasma of patients and healthy individuals and analyzed by Zetasizer Nano ZS to establish their size. The distribution curve of MVs was unimodal and the value of particle dimension (Z) was between 100 and 1000 nm (Fig. 2A), specific to MVs. In addition, the structure and size of MVs isolated from plasma of patients and healthy subjects, were investigated using both fluorescence (Fig. 2B) and transmission electron microscopy (Fig. 2C), to confirm their size and also to characterize their structure. Image analysis showed that, the majority population of vesicles was found in a range of values specific to MVs (100–1000 nm), and these MV fractions contain heterogeneous particles of larger dimensions.

**Fig. 3:**
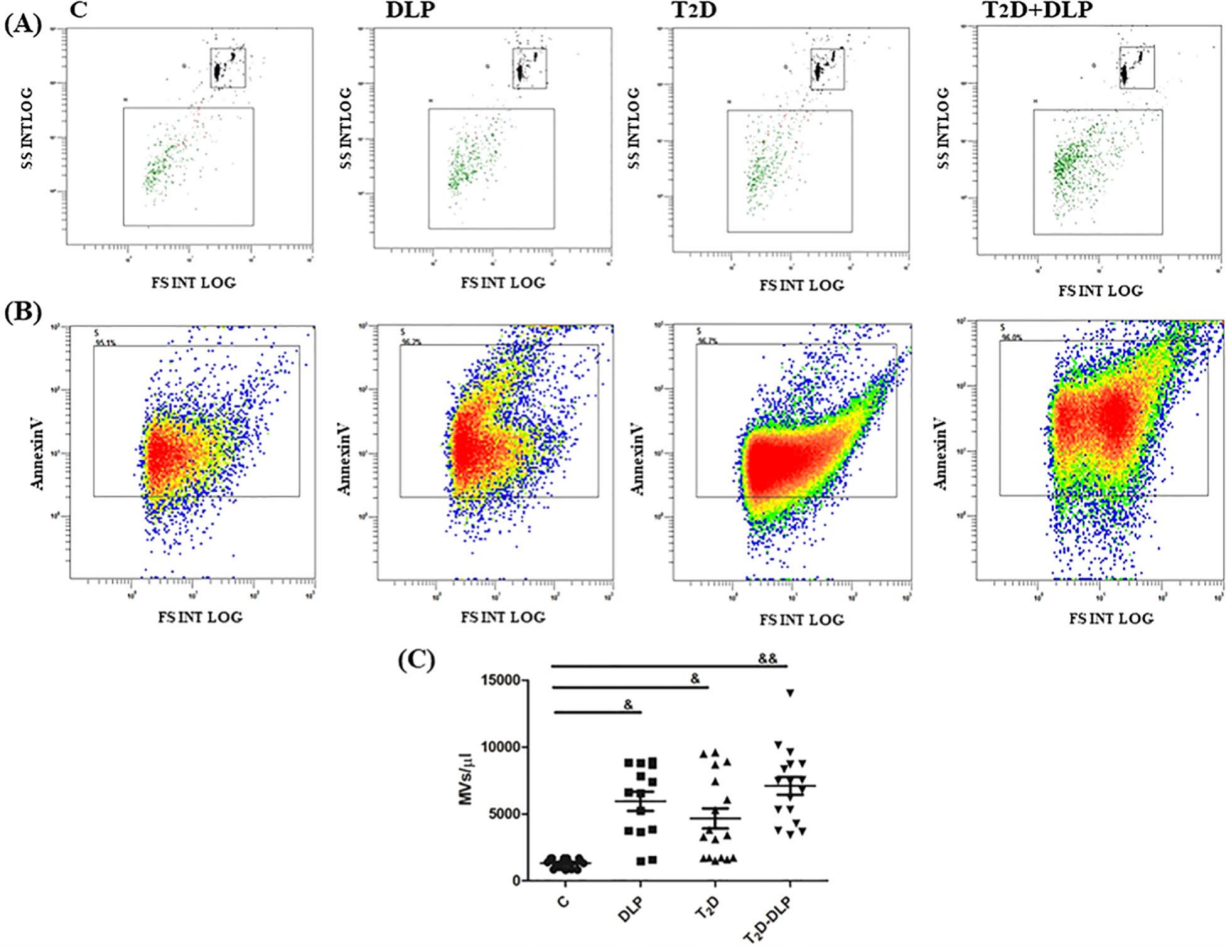
Characterization and quantification of circulating MVs by flow cytometery in plasma from patient groups DLP (n = 14), T_2_D (n = 17), T_2_D-DLP (n = 17) and group C (n = 15) of healthy subjects. Representative dot-plots assigned for MV quantification, based on calibration beads of 10 μm in diameter and 1000/µL concentration (**A**), and MV purity based on the percentages of MVs positive for Annexin V (**B**). Annexin-V-positive plasma MV concentrations for all groups of subjects investigated (**C**). Data are represented as means ± SD. ^&^p < 0.05, ^&&^*p* < 0.01 (vs C) (One-Way ANOVA with Bonferroni’s Multiple Comparison Test)

**Fig. 4:**
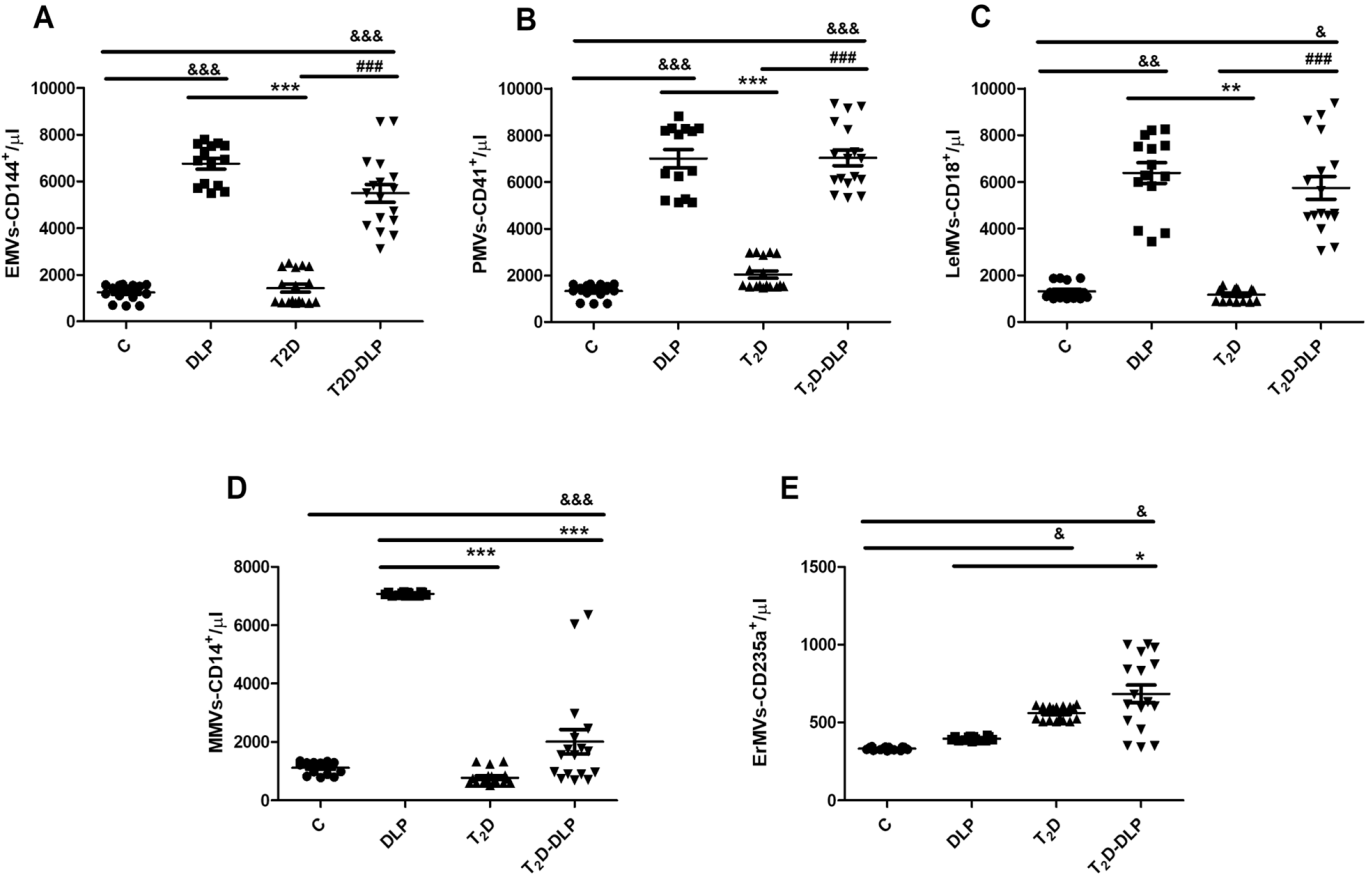
The plasma concentrations of MVs from patient groups DLP (n = 14), T_2_D (n = 17), T_2_D-DLP (n = 17) and group C (n = 15) of healthy subjects, originating from different cell types: MVs derived from endothelial cells, EMVs: AnnexinV^+^/CD144^+^ (**A**), platelets, PMVs: AnnexinV^+^/CD41^+^ (**B**), leucocytes, LeMVs: AnnexinV^+^/CD18^+^ (**C**), monocytes, MMVs: AnnexinV^+^/CD14^+^ (**D**), and erythrocytes, ErMVs: AnnexinV^+^/CD235a^**+**^ (**E**). Data are expressed as means ± SD. ^&^*p* < 0.05, ^&&^*p* < 0.01, ^&&&^*p* < 0.001, (vs C), ^*^p < 0.05, ^**^p < 0.01, ^***^p < 0.001 (vs DLP), ^#^p < 0.05, ^##^p < 0.01, ^###^p < 0.001 (vs T_2_D) (One-Way ANOVA with Bonferroni’s Multiple Comparison Test)

Flow cytometry was used to characterize and quantify the total MVs isolated from plasma of individuals taken into the study, and also to analyze the levels of specific cell derived MVs (Figs. 3, 4). MVs were characterized and quantified by calibration with beads of known size and concentration (1000 beads/µL, 10 µm diameter) (Fig. 3A). In addition, staining of specific marker for MVs, phosphatidylserine (PS), with AnnexinV-FITC, was used to characterize and highlight the purity of MVs isolated from both patient and healthy subject plasma (Fig. 3B).

In patients with diabetic dyslipidemia, as well as in patients with diabetes or dyslipidemia alone, the levels of circulating MVs were significantly higher compared to healthy subjects (Fig. 3C). All these data are sustained by previous reports regarding altered levels of MVs in many diseases including hypercholesterolemia [12], diabetes [38, 39], atherosclerosis [10], cancer [11], which suggest that circulating MVs could be used as biomarkers for the onset and progression of certain disease. It should be noted that our results did not highlight differences in the level of circulating MVs between the groups of patients investigated (Fig. 3C).

Further, the levels of plasma MVs derived from endothelial cells (EMVs: CD144^+^, AnnexinV^+^), platelets (PMVs: CD41^+^, AnnexinV^+^), leukocytes (LeMVs: CD18^+^, AnnexinV^+^), monocytes (MMVs: CD14^+^, AnnexinV^+^), and erythrocytes (ErMVs: CD235a^+^, AnnexinV^+^) were analyzed by flow cytometry for DLP, T_2_D, T_2_D-DLP patient groups and control group. The concentrations of plasma EMVs, PMVs, LeMVs, MMVs and ErMVs were calculated, reporting their percentages for double labeling, to the percentage of 100% of the total plasma MVs (AnnexinV^+^), for which the concentration was previously calculated (Fig. 3) according to the formula presented in materials and methods (Fig. 4).

As shown in Fig. 4, in the case of diabetic patients with dyslipidemia (T_2_D-DLP group) were found significantly increased concentrations of plasma EMVs, PMVs and LeMVs compared to healthy subjects (C group) or diabetic patients (T_2_D group) (Fig. 4A–C), and of plasma ErMVs compared to healthy subjects and dyslipidemic patients (DLP group) (Fig. 4E). Diabetic dyslipidemia, however, did not induce changes in plasma concentrations of MMVs compared to any of the investigated groups (Fig. 4D). It should be noted that, except for MMVs, all other types of plasma MVs investigated, respectively EMVs, PMVs, LeMVs and ErMVs, presented significantly increased levels in patients with diabetic dyslipidemia compared to healthy individuals (Fig. 4). There are no studies that highlight the effect of diabetic dyslipidemia on circulating MV levels or on certain cell types of MVs. Our result could be valuable information in finding new biomarkers for monitoring patients with diabetic dyslipidemia.

Significant changes were also observed when plasma MV levels from diabetic patients (T_2_D group) or patients with dyslipidemia alone (DLP group) were compared to those from healthy subjects (C group). Dyslipidemia was associated with significant increases in plasma concentrations of EMVs, PMVs, LeMVs and MMVs (Fig. 4A–D), while diabetes only with significant increases in plasma concentrations of ErMVs (Fig. 4E). Our results suggest that dyslipidemia and less diabetes generate significant changes in the plasma concentrations of MVs.

Consistent with growing data suggesting that platelet-derived MVs (PMVs) represent over 50% of all circulating MVs, we found that in both diabetic dyslipidemia and dyslipidemia, PMVs are the most abundant in the circulation. Increased PMV levels were found in numerous diseases such as diabetes mellitus, cardiovascular diseases, coagulation disorders, rheumatoid arthritis, cancers, infections and atherosclerosis [29, 40–42], as main regulators of inflammation, hemostasis and angiogenesis [29, 41, 42]. Patients with hypercholesterolemia displayed elevated levels of PMVs and MMVs compared to healthy subjects Moreover, in familial hypercholesterolemia, increased concentrations of EMVs, PMVs, LeMVs, MMVs and ErMVs have been found [43].

Although in our study we found that type 2 diabetes induces changes only in the level of plasma concentrations of total MVs and ErMVs, the other types of vesicles investigated having normal values, there are still studies that showed that AnnexinV-positive blood cell microvesicles (total MVs) and particularly EMVs, PMVs and MMVs had higher levels in patients with either type 1 or type 2 diabetes [44] compared with healthy individuals. According to our results, [45] Freeman et al. found that ErMV levels were significantly higher in individuals with diabetes compared with controls [45], while Giannella et al. [13] showed that LeMV levels were not significantly different between T_2_D patients and controls [46] or pre-diabetic (PreDM) patients. Also, PreDM and T_2_D patients showed significantly higher levels of circulating activated endothelial cell-derived MVs (AnnexinV^+^/CD62E^+^) compared to normal glucose tolerance (NGT) subjects, with the specification that the highest levels were recorded in the case of T_2_D patients [13, 44]. Moreover, in vitro it was demonstrated that endothelial cells exposed to high glucose can alter EMV content and also induce modifications of cellular function [47]. Also, more data confirmed that the levels of circulating PMVs were increased in diabetic patients compared with controls [48–51], but after treatment with anti-platelet drugs cilostazol or sarpogrelate hydrochloride their number was significantly decreased [49–51]. It has been showed that drugs used in prevention of cardiovascular disease also affect the number and composition of PMVs [51–53]. Moreover, patients treated with statins registered lower numbers of extracellular vesicles, especially of those derived from platelets, leukocyte and endothelial cells, compared to untreated patients [53, 54]. However, all these data are inconsistent, since extracellular vesicle concentration often depends on the circumstance of their release, trigger, molecular cargo, or cells of origin [53].

Finally, Hototen and Evans concluded that the levels of circulating MVs, in particular those derived from monocytes, platelets, and endothelial cells, could be used as a biomarker for T_2_D patients, but considering the treatment plan followed and the onset of disease for each patient. Regarding diabetic dyslipidemia, we can assume that MV concentration might be dependent by the presence of either dyslipidemia (EMVs, PMVs and LeMVs) or diabetes (ErMVs) for each type of MV we investigated.

Furthermore, significant Pearson correlations between circulating MVs (EMVs, PMVs, LeMVs, MMVs, ErMVs) and metabolic parameters or plasma inflammatory cytokines in plasma from diabetic patients with dyslipidemia investigated in our study, are shown in Tables 3 and 4. Positive correlations between PMVs and HbA1c (r = 0.90, p = 0.01), MMVs and ALT (r = 0.96, p = 0.01) or leucocytes (r = 0.91, p = 0.03), and between total MVs and TG (r = 0.92, p = 0.02) or urea (r = 0.86, p = 0.05) can be noticed (Table 3). In addition, negative correlations between MMVs and monocytes (r = 0.94, p = 0.01) and between total MVs and HDL-c (r = 0.91, p = 0.03) can be observed (Table 3). A positive correlation was also observed between MMVs and inflammatory factor IFNγ (r = 0.86, p = 0.05) in diabetic patients with dyslipidemia (Table 4). Until now, there are no studies that highlight the possible correlations that appear in diabetic dyslipidemia between plasma MVs and metabolic parameters or plasma inflammatory markers. There are studies that emphasize the presence of certain correlations only in diabetes or dyslipidemia. Thus, in diabetic patients, PMVs and EMVs were inversely related to HbA1c [53, 55]. Also, Gianella et al. [13] found a negative correlation between CD62E^+^MVs and plasma glucose of PreDM and NGT individuals. In familial hypercholesterolemia, the levels of circulating MMVs were directly correlated with oxLDL plasma concentrations [54].

**Table 3:** Significant correlations among circulating/plasma MVs and relevant clinical parameters in the diabetic dyslipidemia context

T_2_D-DLP group		ErMVs	PMVs	LeMVs	MMVs	EMVs	Total MVs
Clinical measurements ↓	n	17	17	17	17	17	17
TC	r	0.552	0.094	0.300	− 0.421	0.255	**0.822**
	P	0.332	0.880	0.613	0.463	0.674	**0.081**
HDL-c	r	− 0.484	− 0.102	− 0.442	0.302	− 0.163	− ***0.911***
	P	0.413	0.863	0.451	0.624	0.791	***0.030***
LDL-c	r	0.493	0.553	− 0.632	0.624	0.614	0.101
	P	0.394	0.334	0.252	0.262	0.272	0.873
TG	r	− 0.053	− 0.313	**0.812**	− 0.261	− 0.380	***0.924***
	P	0.933	0.603	**0.091**	0.672	0.521	***0.022***
TGO	r	− 0.314	− 0.453	**0.820**	− 0.093	− 0.583	**0.801**
	P	0.605	0.441	**0.080**	0.873	0.302	**0.103**
TGP	r	0.013	0.350	− 0.643	***0.964***	0.261	− 0.201
	P	0.985	0.560	0.244	***0.013***	0.663	0.742
Fasting Glucose	r	− 0.703	− 0.391	− 0.164	**0.811**	− 0.494	− 0.100
	P	0.184	0.512	0.793	**0.094**	0.392	0.871
HbA1c	r	0.761	***0.983***	− 0.564	0.222	***0.870***	− 0.382
	P	0.134	***0.014***	0.313	0.711	***0.052***	0.524
Hemoglobin	r	− 0.652	− 0.433	0.512	0.323	− 0.691	0.212
	P	0.224	0.464	0.372	0.593	0.193	0.733
Creatinine	r	− 0.702	− 0.624	0.464	0.402	− 0.75	0.440
	P	0.170	0.262	0.432	0.503	0.142	0.450
Urea	r	− 0.261	− 0.433	0.633	0.083	− 0.494	***0.861***
	P	0.663	0.461	0.251	0.894	0.392	***0.053***
Uric acid	r	0.483	0.114	0.631	− 0.582	0.111	**0.814**
	P	0.403	0.851	0.240	0.293	0.853	**0.092**
Erythrocytes	r	0.693	0.352	0.394	− 0.452	0.371	0.731
	P	0.192	0.560	0.513	0.431	0.533	0.152
Platelets	r	0.253	0.252	− **0.803**	0.242	0.484	− 0.443
	P	0.672	0.670	**0.094**	0.692	0.400	0.454
Leucocytes	r	− 0.422	0.063	− 0.583	***0.911***	− 0.071	− 0.601
	P	0.470	0.912	0.302	***0.030***	0.892	0.272
Monocytes	r	0.091	− 0.251	0.421	− ***0.940***	− 0.091	− 0.051
	P	0.874	0.682	0.482	***0.011***	0.873	0.920
SBP	r	0.423	0.624	0.021	− 0.122	0.393	− 0.250
	P	0.474	0.261	0.960	0.833	0.502	0.681
DBP	r	− 0.773	− 0.710	0.470	0.363	− **0.811**	0.422
	P	0.124	0.171	0.421	0.554	**0.092**	0.474

Spearman rank correlation test was used to assess relationships among variables. Bolded coefficients show a powerful correlation, and of them only italic underlined coefficients are significant (p < 0.05)

**Table 4:** Significant correlations among circulating/plsasma inflammatory cytokines and circulating MVs in the diabetic dyslipidemia context

T_2_D-DLP group		IFNγ	TNFα	IL1β	IL6
Plasma MVs↓	n	17	17	17	17
ErMVs	r	0.034	− 0.301	0.132	− 0.181
	P	0.961	0.613	0.831	0.760
PMVs	r	0.510	− 0.332	0.260	0.011
	P	0.362	0.581	0.660	0.973
LeMVs	r	− **0.801**	− 0.470	0.311	0.222
	P	**0.102**	0.421	0.603	0.714
MMVs	r	***0.861***	0.173	− 0.371	0.561
	P	***0.050***	0.781	0.531	0.322
EMVs	r	0.383	− 0.101	0.050	− 0.213
	P	0.522	0.870	0.931	0.720
total MVs	r	− 0.681	− 0.372	− 0.223	0.381
	P	0.201	0.533	0.711	0.521

Spearman rank correlation test was used to assess relationships among variables. Bolded coefficients show a powerful correlation, and of them only italic underlined coefficients are significant (p < 0.05)

### Microvesicle-associated and circulating microRNAs in diabetic dyslipidemia

#### MicroRNA expression profile in plasma microvesicles

**Fig. 5:**
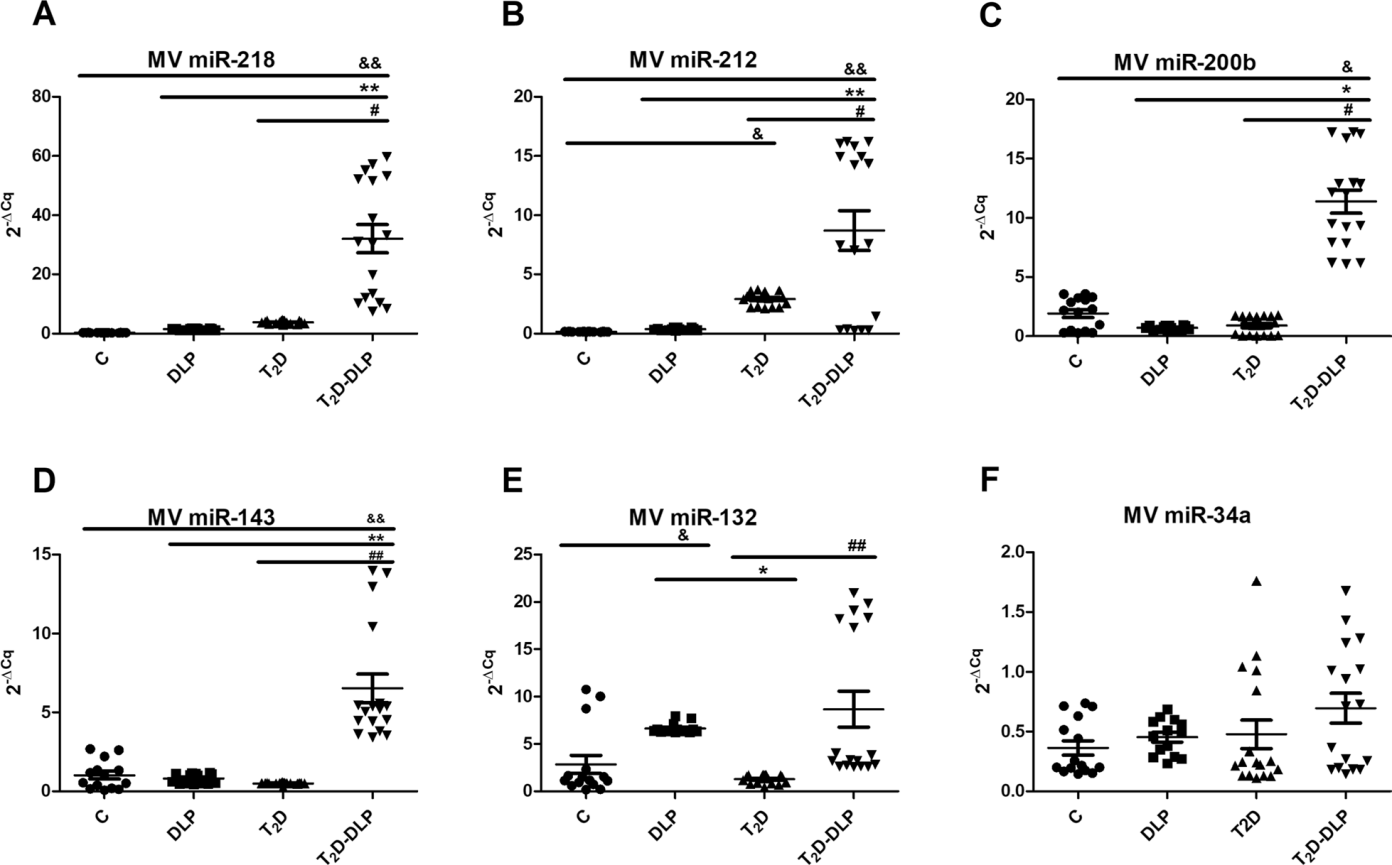
Relative expression (2^−ΔCq^, ΔCq = Cq has-miRNA—Cq U6) of miRNAs in MVs from the plasma of patients with dyslipidemia (n = 14), diabetes (n = 17), diabetes associated with dyslipidemia (n = 17) and of healthy subjects taken as control (n = 15): **A** miR-218-5p; **B** miR-212-5p; **C** miR-200b-3p; **D** miR-143-3p; **E** miR-132-3p; **F** miR-34a-5p. Data are expressed by means ± SD. ^&^*p* < 0.05 (vs C), *p < 0.05 (vs DLP), ^#^*p* < 0.05 (vs T_2_D), ^&&/**/##^*p* < 0.01 (One-Way ANOVA with Bonferroni's Multiple Comparison Test)

**Fig. 6:**
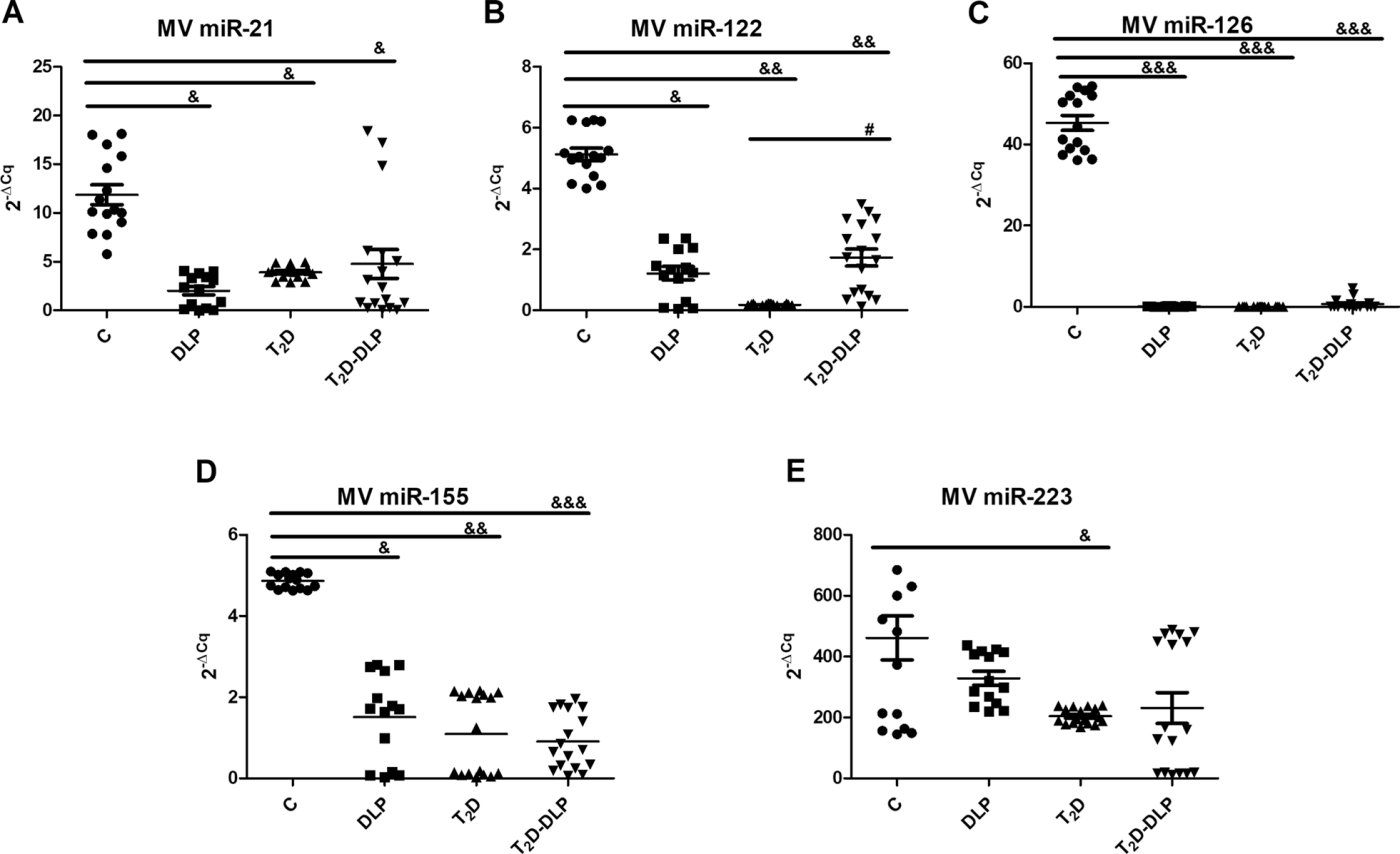
Relative expression (2^−ΔCq^, ΔCq = Cq has-miRNA—Cq U6) of miRNAs in MVs from the plasma of patients with dyslipidemia (n = 14), diabetes (n = 17), diabetes associated with dyslipidemia (n = 17) and of healthy subjects taken as control (n = 15): **A** miR-21-5p; **B** miR-122-5p; **C** miR 126-3p; **D** miR-155-5p; **E** miR-223-3p. Data are expressed as means ± SD. ^&^*p* < 0.05, ^&&^*p* < 0.01, ^&&&^*p* < 0.001 (vs C), ^#^*p* < 0.05 (vs T_2_D) (One-Way ANOVA with Bonferroni's Multiple Comparison Test)

Original studies, indicated that extracellular vesicles are important mediators of intercellular communication due to their RNA content, particularly miRNAs, which regulates transcription and translation of several genes [22, 56, 57]. Recently, in a study focused on the relationships between miRNA polymorphisms, insulin signaling pathways and T_2_D, it was reported that miR-133a-2 rs13040413, let-7a-1 rs13293512 and miR-27a rs895819 may be associated with T_2_D susceptibility [58]. Common genetic and epigenetic aspects in T_2_D and cardiovascular disease, including microRNAs and long non-coding RNAs, whose aberrant regulation has been implicated in both disease states, either etiologically or as a cause of their progression, have been discussed by [59]. Furthermore, a growing number of studies has shown that a specific set of miRNAs has an altered expression profile in both the progression of diabetes and dyslipidemia, making these biomolecules potential biomarkers for disease prognosis, diagnosis, and management [21, 22]. They have been reported as biomarkers and due to the beneficial effects that certain drugs such as empagliflozin [60] or physical exercises [61] have on them in patients with heart failure with preserved ejection fraction and diabetes or patients with diabetic heart. Knowing also that, modulation of miRNA expression affects insulin production and the incidence of vascular complications in diabetes [21, 22], in our study we analyzed the expression of 11 miRNAs, from those previously reported to undergo changes during diabetes and dyslipidemia evolution. Selected miRNAs, miR-21-5p, -34a-5p, -122-5p, -126-3p, -132-3p, -143-3p, -155-5p, -200b-3p, -212-5p, -218-5p and -223-3p, were first investigated in MVs isolated from plasma of patients with diabetic dyslipidemia, with diabetes or dyslipidemia alone and of healthy subjects (Figs. 5 and 6). However, a systematic investigation of microvesicle-associated microRNAs (MV miRNAs) in diabetic dyslipidemic patients has not yet been reported to our knowledge.

In diabetic patients with dyslipidemia (T_2_D-DLP group), we found significantly elevated levels of miR-218, miR-212, miR-200b and miR-143 contained in circulating MVs, compared to all the other experimental groups (Fig. 5:A–D). Also, in diabetic dyslipidemia, an up-regulation of miR-132 and miR-122, contained in circulating MVs, was observed when compared to T_2_D group (Figs. 5E, 6B). It should be noted that, the miR-132 also registered strong negative correlations with plasma levels of cholesterol (r = 0.86, p = 0.05), uric acid (r = 0.89, p = 0.01) and erythrocytes (r = 0.95, p = 0.01) (Table 5). In the case of miR-34a, a slightly increasing trend was observed for investigated patients compared to healthy subjects, but not statistically significant (Fig. 5F).

**Table 5:** Significant correlations among microvesicle-associated microRNAs and relevant clinical parameters in the diabetic dyslipidemia context

T_2_D-DLP group		MV miR-21	MV miR-34a	MV miR-132	MV miR-143	MV miR-223	MV miR-122	MV miR-126	MV miR-155	MV miR-200b	MV miR-212	MV miR-218
Clinical measurements ↓	n	17	17	17	17	17	17	17	17	17	17	17
TC	r	***0.901***	− 0.751	− ***0.861***	− 0.711	0.012	− 0.271	− 0.091	***0.881***	− 0.531	0.4002	− 0.771
	P	***0.031***	0.144	***0.050***	0.171	0.991	0.650	0.882	***0.040***	0.352	0.494	0.123
HDL-c	r	− 0.692	0.753	**0.831**	0.752	0.150	0.254	0.051	− ***0.902***	0.531	− 0.292	0.661
	P	0.181	0.141	**0.072**	0.133	0.800	0.681	0.920	***0.032***	0.322	0.634	0.212
LDL-c	r	0.331	− 0.470	− 0.182	− **0.821**	0.631	− ***0.992***	0.064	0.011	0.183	− 0.562	− 0.434
	P	0.570	0.412	0.762	**0.080**	0.251	***0.0014***	0.910	0.974	0.764	0.321	0.464
TG	r	0.272	− 0.624	− 0.471	− 0.321	− 0.372	0.142	− 0.301	**0.853**	− 0.762	0.440	− 0.133
	P	0.651	0.252	0.412	0.583	0.531	0.811	0.620	**0.062**	0.134	0.440	0.822
TGO	r	0.022	− 0.522	− 0.190	− 0.120	− 0.334	0.213	− 0.414	0.681	− 0.753	0.404	0.161
	P	0.972	0.363	0.754	0.831	0.571	0.723	0.492	0.194	0.144	0.502	0.790
TGP	r	− 0.242	− 0.191	0.370	− 0.421	0.624	− **0.831**	− 0.050	− 0.371	0.301	− 0.740	0.151
	P	0.681	0.751	0.52	0.471	0.251	**0.081**	0.932	0.522	0.611	0.142	0.790
Fasting Glucose	r	− 0.431	− 0.221	0.771	0.172	0.570	− 0.322	− 0.600	− 0.292	− 0.152	− 0.223	0.750
	P	0.451	0.722	0.124	0.770	0.300	0.580	0.273	0.631	0.804	0.721	0.140
HbA1c	r	− 0.191	0.430	− 0.302	− 0.493	− 0.222	− 0.361	***0.913***	− 0.382	**0.823**	− **0.821**	− 0.512
	P	0.752	0.463	0.614	0.391	0.711	0.542	***0.021***	0.513	**0.082**	**0.082**	0.373
Hemoglobin	r	− 0.620	− 0.021	0.422	0.282	− 0.260	0.281	− 0.323	0.031	− 0.322	0.014	0.681
	P	0.263	0.962	0.471	0.642	0.662	0.642	0.591	0.963	0.591	0.992	0.190
Creatinine	r	− 0.252	− 0.471	0.402	0.111	0.150	0.010	− 0.704	0.261	− 0.620	0.194	0.621
	P	0.683	0.420	0.502	0.850	0.803	0.991	0.182	0.672	0.251	0.742	0.2503
Urea	r	0.221	− 0.782	− 0.181	− 0.323	0.010	− 0.102	− 0.573	0.734	− **0.810**	0.350	0.080
	P	0.714	0.112	0.762	0.594	0.981	0.862	0.304	0.151	**0.090**	0.553	0.881
Uric acid	r	0.522	− 0.433	− ***0.890***	− 0.521	− 0.560	0.141	0.172	**0.852**	− 0.462	0.360	− 0.60
	P	0.364	0.461	***0.042***	0.362	0.320	0.821	0.780	**0.060**	0.420	0.543	0.234
Erythrocytes	r	0.592	− 0.440	− ***0.950***	− 0.720	− 0.422	− 0.100	0.303	0.770	− 0.292	0.160	− 0.180
	P	0.281	0.441	***0.012***	0.162	0.470	0.862	0.611	0.122	0.630	0.794	0.092
Platelets	r	0.360	− 0.034	0.133	− 0.142	0.761	− 0.560	− 0.023	− 0.350	0.353	− 0.211	− 0.230
	P	0.540	0.942	0.821	0.810	0.131	0.312	0.961	0.552	0.554	0.722	0.700
Leucocytes	r	− 0.691	0.272	**0.833**	0.203	0.484	− 0.370	− 0.100	− 0.760	0.444	− 0.664	0.653
	P	0.192	0.653	**0.073**	0.731	0.412	0.534	0.870	0.131	0.451	0.224	0.231
Monocytes	r	0.353	0.210	− 0.371	0.390	− 0.454	0.702	0.091	0.273	− 0.172	0.683	− 0.250
		0.551	0.721	0.533	0.512	0.442	0.184	0.88	0.654	0.771	0.192	0.671
SBP	r	− 0.492	0.654	− 0.264	− 0.071	− **0.781**	0.284	***0.870***	− 0.253	0.582	− 0.490	− 0.210
	P	0.401	0.232	0.662	0.911	**0.110**	0.642	***0.053***	0.674	0.291	0.390	0.731
DBP	r	− 0.220	− 0.473	0.441	0.190	0.200	0.041	− 0.781	0.254	− 0.660	0.281	0.672
	P	0.713	0.421	0.440	0.752	0.741	0.941	0.112	0.673	0.213	0.640	0.21

Spearman rank correlation test was used to assess relationships among variables. Bolded coefficients show a powerful correlation, and of them only italic underlined coefficients are significant (p < 0.05)

The other investigated MV miRNAs, respectively miR-21, miR-122, miR-126, miR-155, had the expression levels significantly reduced for all studied patient groups compared to the control group (Fig. 6A, C, D), while down-regulation of miR-223 was not significant for dyslipidemic and diabetic dyslipidemic groups (Fig. 6E). Importantly, these latter MV miRNAs, except for miR-122, did not have altered gene expression levels between the groups of patients (Fig. 6A, C, D, E).

The possible correlations between miRNAs carried by plasma MVs and clinical (Table 5) or inflammatory (Table 6) parameters in diabetic dyslipidemia were established. Anywise, MV miR-21 and MV miR-155 displayed positive correlations with plasma levels of cholesterol (r = 0.90, p = 0.03, respectively r = 0.88, p = 0.04), MV miR-126 with HbA1c levels (r = 0.91, p = 0,02) and SBP (r = 0.87, p = 0.05) (Table 5), MV miR-155 presented negative associations with plasma levels of HDL-c (r = 0.90, p = 0.03) and MV miR-122 only with LDL-c levels (r = 0.99, p = 0.001) (Table 5). In addition, negative correlations were recorded for MV miR-223 gene expression with IL-1β plasma levels (r = 0.92, p = 0.02) and MV miR-212 with IFNγ levels (r = 0.92, p = 0.02) (Table 6).

**Table 6:** Significant correlations among microvesicle-associated microRNAs and circulating/plasma inflammatory cytokines in the diabetic dyslipidemia context

T_2_D-DLP group		IFNγ	TNFα	IL-1β	IL-6
MV miRNAs ↓	n	17	17	17	17
MV miR-21	r	− 0.591	0.234	− 0.541	− 0.351
	P	0.292	0.701	0.342	0.551
MV miR-34a	r	0.383	− 0.090	− 0.141	0.092
	P	0.521	0.871	0.161	0.604
MV miR-132	r	0.530	0.474	**0.822**	− 0.204
	P	0.341	0.414	**0.811**	0.873
MV miR143	r	− 0.090	0.311	0.280	− 0.330
	P	0.881	0.602	0.631	0.571
MV miR-223	r	0.370	0.791	− ***0.921***	− 0.070
	P	0.531	0.103	***0.020***	0.902
MV miR-122	r	− 0.540	− 0.221	− 0.662	− 0.270
	P	0.342	0.710	0.220	0.651
MV miR-126	r	0.311	− 0.502	0.683	− 0.123
	P	0.602	0.390	0.203	0.833
MV miR-155	r	− **0.803**	− 0.301	− 0.192	0.161
	P	**0.091**	0.612	0.741	0.790
MV miR-200b	r	0.742	− 0.011	0.384	− 0.202
	P	0.141	0.980	0.524	0.741
MV miR-212	r	− ***0.924***	0.183	− 0.151	− 0.330
	P	***0.021***	0.772	0.801	0.583
MV miR-218	r	0.261	0.242	0.011	0.254
	P	0.671	0.691	0.980	0.681

Spearman rank correlation test was used to assess relationships among variables. Bolded coefficients show a powerful correlation, and of them only italic underlined coefficients are significant (p < 0.05)

Importantly, we tried to find possible correlations between MV miRNA expressions, levels of different types of MVs in plasma and total circulating MVs levels. The analysis revealed the presence of some positive correlations between MV miR-126 and the levels of PMVs (r = 0.86, p = 0.05), MV miR-155 and total levels of MVs (r = 0.97, p = 0.01), and also of some negative correlations between MV miR-218 and the levels of ErMVs (r = 0.94, p = 0.01) (Table 7).

**Table 7:** Significant correlations among microvesicle-associated microRNAs (MV miRNAs) and circulating/plasma MVs in the diabetic dyslipidemia context

T_2_D-DLP group		ErMVs	PMVs	LeMVs	MMVs	EMVs	Total MVs
MV miRNAs ↓	n	17	17	17	17	17	17
MV miR-21	r	0.461	− 0.012	0.100	0.430	0.261	0.603
	P	0.422	0.981	0.874	0.461	0.673	0.271
MV miR-34a	r	− 0.024	0.291	− 0.263	− 0.053	0.152	− **0.822**
	P	0.973	0.630	0.662	0.922	0.793	**0.081**
MV miR-132	r	− 0.791	− 0.403	− 0.273	0.580	− 0.492	− 0.600
	P	0.100	0.494	0.642	0.292	0.393	0.271
MV miR143	r	− 0.751	− 0.613	0.221	− 0.190	− 0.651	− 0.470
	P	0.140	0.262	0.71	0.752	0.224	0.412
MV miR-223	r	− 0.162	− 0.110	− 0.590	0.603	0.051	− 0.130
	P	0.780	0.852	0.281	0.281	0.934	0.833
MV miR-122	r	− 0.401	− 0.480	0.672	− 0.683	− 0.552	− 0.040
	P	0.503	0.404	0.211	0.202	0.330	0.941
MV miR-126	r	0.720	***0.863***	− 0.330	− 0.124	0.761	− 0.390
	P	0.161	***0.050***	0.581	0.842	0.133	0.503
MV miR-155	r	0.173	− 0.284	0.693	− 0.514	− 0.182	***0.971***
	P	0.783	0.633	0.193	0.372	0.75	***0.012***
MV miR-200b	r	0.394	0.751	− 0.754	0.313	0.671	− **0.841**
	P	0.500	0.133	0.132	0.601	0.212	**0.072**
MV miR-212	r	− 0.351	− 0.780	0.740	− 0.711	− 0.620	0.552
	P	0.553	0.112	0.143	0.170	0.262	0.331
MV miR-218	r	− ***0.944***	− 0.631	0.121	0.392	− 0.770	− 0.330
	P	***0.014***	0.241	0.844	0.510	0.124	0.571

Spearman rank correlation test was used to assess relationships among variables. Bolded coefficients show a powerful correlation, and of them only italic underlined coefficients are significant (p < 0.05)

Many studies have been performed on exosomes carrying several types of miRNAs and only a few attested the role of MVs in caring regulator miRNAs in diabetes or dyslipidemia.

So far, little is known about miR-218, miR-143 and miR-34a contained in EVs in diabetes. Higher amounts of miR-218 were found in urinary exosomes from T_1_D children, compared to healthy controls [62]. Exosomal miR-34a has been found increased in T_1_D and dyslipidemic patients being associated with alterations of lipid metabolism (increased levels of cholesterol and triglycerides) and endothelial function [63]. We have previously showed that MVs released from platelets isolated from hypertensive-hyperlipidemic hamsters, contain significantly increased levels of miR-34a and reduced of miR-143 [29], platelets being considered the main source of extracellular vesicles in plasma [22, 64]. In contradiction, we found here that MVs obtained from patients with diabetes or dyslipidemia alone, carry, just like MVs from healthy subjects, decreased levels of miR-218, miR-143 and miR-34a, but MV miR-218 and MV miR-143 levels were significantly augmented in diabetic patients with dyslipidemia (Fig. 5A, D, F).

EVs derived from beta cells also hold the miRNA profile of the host cells, including miR-212 and miR-132 [65, 66]. In mature pancreatic beta cells miR-212 and miR-132 are key players in insulin secretion [65, 66]. It is believed that miR-132 might have a protecting role against diabetes development by promoting cell growth and reducing cell death in beta cells [67], down-regulated miR-132 being associated with a minor inhibition of cell proliferation [68]. However, contradictory data attested up-regulation of miR-132 expression in several T_2_D models [68]. Furthermore, mir-132 has been found increased in both EVs in early gestation and placenta exosomes in gestational diabetes patients [69], while in plasma from second-trimester gestational diabetes patients was insignificantly [70]. Two other studies performed on a large group of patients found miR-132 decreased in gestational diabetes pregnancies [71, 72]. In line with these results, we also found a slightly but not significant decrease of miR-132 levels in MVs isolated from T_2_D patients compared to healthy individuals (Figs. 4, 5E). Our results also highlighted significantly increased levels of miR-132 in MVs from patients with dyslipidemia, compared to control subjects (Fig. 5E), being in agreement with a study that showed that in metabolic syndrome EVs derived from porcine adipose derived stem cells have been found to contain high levels of miR-132 [73]. In our study the levels of miR-212 have been found significantly increased only in MVs from patients with T_2_D alone, compared to control subjects, while in the group of the patients with dyslipidemia MV miR-212 was not significantly modified (Fig. 5B).

In conclusion, from our results we can infer that only the association of dyslipidemia with diabetes could significantly increase the gene expression levels of miR-218, miR-212, miR-200b, miR-143, and miR-132 contained in plasma MVs (Fig. 5).

Regarding the MV miRNAs that we found to be down-regulated in diabetic dyslipidemia, there are some studies that confirm our results, while others deny them. Thus, circulating EV miR-21 was considered to be a possible biomarker for T_1_D progression, both in vitro and in vivo studies reporting increased levels of miR-21 transported by EVs. Therefore, up-regulated miR-21 was found in MIN6 beta cell-line, after treatment with inflammatory cytokines and even before hyperglycemia establishment, in NOD mice. In preclinical studies using hamsters with hyperlipidemia and hypertension, expression of miR-21 was found up-regulated in MVs derived from platelets [29]. Moreover, the increased presence of miR-21 has been reported in children with new-onset T_1_D [74]. Contrary to these results, in our study we found significantly decreased levels of circulating miR-21 carried by MVs in diabetic, dyslipidemic and diabetic dyslipidemic patients (Fig. 6A).

In T_1_D patients [75] and even in the new-onset T_1_D cohort [76], another highly overexpressed EV miRNA was miR-122. In the case of exosomes from obese mice, miR-122 was found increased, while these exosomes led to insulin resistance and glucose intolerance when injected in lean mice [77]. Significantly higher levels of miR-122 have been found in placenta of gestational diabetes patients [78]. In contrary, we found down-regulated expression of miR-122 transported by circulating MVs, in T_2_D compared to control group (Fig. 6B). Even thou medications alter some miRNA cargo in EVs, it has been demonstrated in a very recent study that levels of EV-encapsulated miR-122 were not affected by metformin treatment. Consistent with our results, in metformin-insensitive T_2_D patients, Peng and collaborators found low levels of exosome miRNA-122, indicating miR-122 as biomarker of metformin insensitivity [79]. As important regulator of glucose and lipid metabolism as well [80–82], increased miR-122 showed a specific correlation with monounsaturated and saturated fatty acids within the lipid classes triacylglycerols and cholesterol esters. Also, one year atorvastatin treatment led to significant reduction in both cholesterol and serum miR-122 levels [83]. Contrary to these reports, we found MV miR-122 levels decreased in dyslipidemia alone or diabetic dyslipidemia when compared to healthy individuals (Fig. 6B).

Two others important MV miRNAs investigated by us, which were found in abundance both in platelets and in MVs derived from them [22, 40, 84–87], were miR-126 and miR-223. In addition to platelets, endothelial cells have been found to express miR-126 and, moreover, it has been shown that the highest amount of circulating miR-126 is found in MVs derived from endothelial cells [22, 84].

Previous studies revealed decreased expression of MV miR-126 in patients with diabetes and prevalent cardiovascular disease [22, 88] and miR-223 within MVs released by platelets from hypertensive-hyperlipidemic hamsters [29]. Our data confirm findings attesting miR-126 as one of the most down-regulated MV miRNA, especially in endothelial cells—derived MVs and in the whole plasma [89–91]. Specifically, we found that, the gene expression for MV miR-126 was significantly reduced for all groups of patients included in the study (Fig. 6C), while for MV miR-223 it was significantly reduced only for the group of diabetic patients (Fig. 6E).

Another miRNA found significantly decreased in MVs isolated from patients with diabetic dyslipidemia, with diabetes or dyslipidemia alone, was miR-155 (Fig. 6D). In addition to our results, other clinical studies measuring miR-155 in EVs derived from different sources, pointed out that patients with T_1_D exhibited lower amounts of miR-155, also correlated with the severity of the diseases. Decreased levels of miR-155 in EVs isolated from blood were also found at patients with diabetic retinopathy compared to diabetic patients with no eye defects or to healthy individuals [92, 93]. Also, urinary exosomes from diabetic patients carry lower amount of miR-155, decreased miR-155 being associated with micro albuminuria in T_1_D patients [92, 94].

To conclude, we believe that cumulative effect of both diabetes and dyslipidemia, induced MV modifications leading to up-regulation of miR-218, miR-212, miR-200b and miR-143 and down-regulation of miR-21, miR-122, miR-126 and miR-155 carried by MVs in diabetic dyslipidemia. It is important to point out that the same MV miRNAs showed significant correlations with important plasma markers, representative of this pathology. Thus, miR-218 was negatively correlated with the levels of ErMVs, whereas miR-21 was positively linked with cholesterol plasma levels, miR-122 was negatively associated with LDL-c levels, miR-126 was positively connected with HbA1c and PMV levels, and miR-155 was positively related with cholesterol and total MV levels and in the same time negatively connected with HDL-c levels.

### MicroRNA expression profile in plasma

Since serum or plasma miRNAs are considered potential diagnostic biomarkers in diabetes [95–97] the same miRNAs detected above in plasma MVs have been investigated in circulation. However, there are only few studies to investigate the circulating miRNAs in diabetic dyslipidemia.

**Fig. 7:**
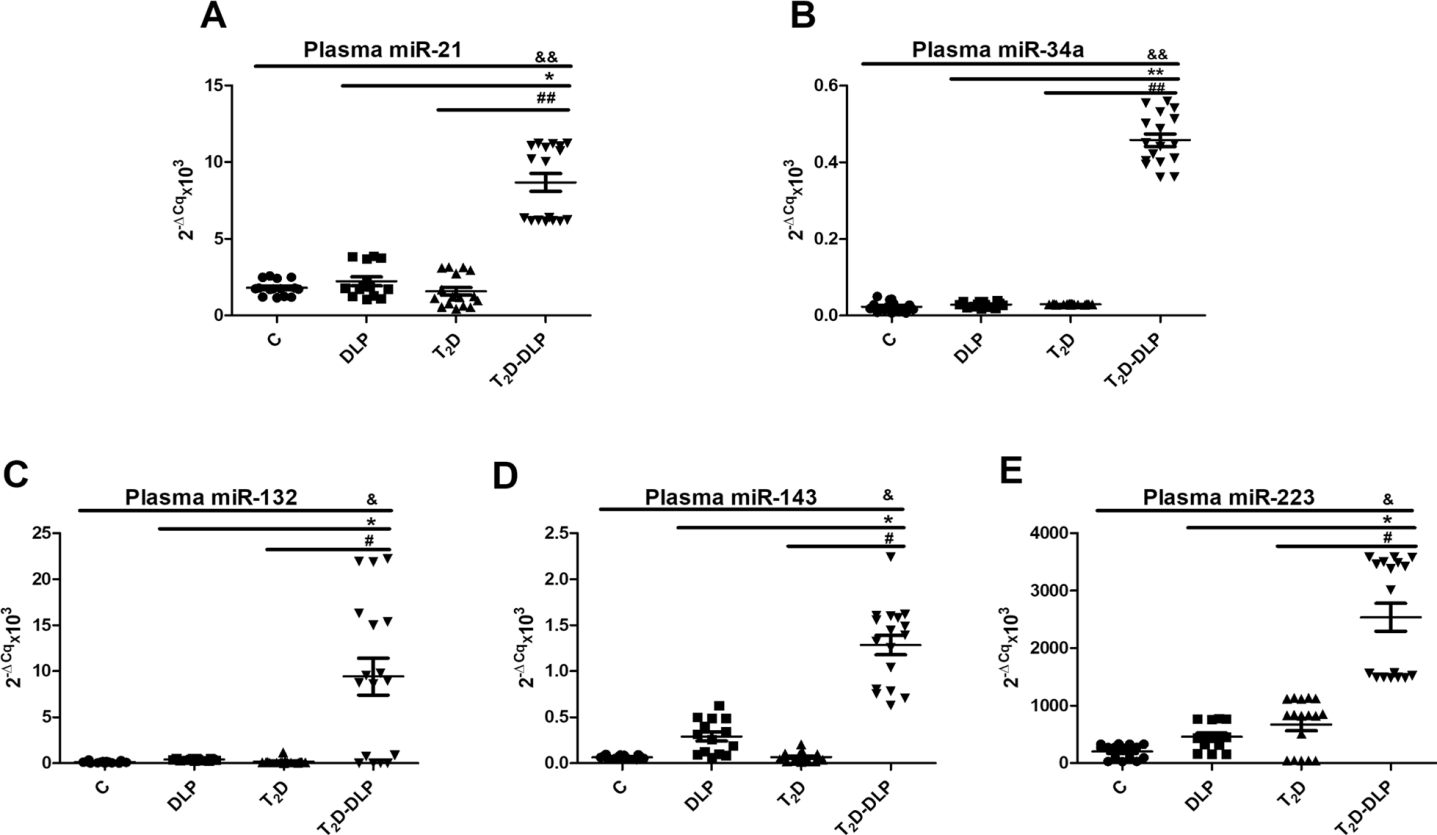
Relative expression (2^−ΔCq^ × 10^3^, ΔCq = Cq has-miRNA—Cq cel-miR-39) of miRNAs in plasma from patients with dyslipidemia (n = 14), diabetes (n = 17), diabetes associated with dyslipidemia (n = 17) and of healthy subjects taken as control (n = 15): **A** miR-21-5p; **B** miR-34a-5p; **C** miR 132-3p; **D** miR-143-3p; **E** miR-223-3p. Data are expressed as means ± SD. ^&^*p* < 0.05 (vs C), *p < 0.05 (vs DLP), ^#^*p* < 0.05 (vs T_2_D), ^&&/**/##^*p* < 0.01 (One-Way ANOVA with Bonferroni's Multiple Comparison Test)

Therefore, we measured the levels of miR-21, miR-34a, miR-132, miR-143, miR-223, and we found them significantly up-regulated in plasma from T_2_D patients with dyslipidemia compared to all the other experimental groups (Fig. 7A–E). Although our results did not show any significant changes in the expressions of these miRNAs neither in diabetes nor in dyslipidemia, we believe that the significantly increased miRNA plasma levels found in diabetes associated with dyslipidemia could be the result of cumulative effect of these two diseases.

MiR-21, known as a key player of cell proliferation and apoptosis, was frequently up-regulated in many types of diseases, being considered diagnostic marker and therapeutic modulator for T_2_D [98]. Circulating miR-21 may act as powerful indicator even for an early detection of glucose adjustments in pre-diabetes stages [99]. Previous data highlighted the idea that plasma miR-21 is up-regulated in pre-diabetes [99], in T_2_D [99, 100] and in proliferative diabetic retinopathy [100]. Levels of miR-21 have been linked with clinical metabolic parameters characteristic for obesity, BMI and waist circumference [101]. We have also found a slightly but not significant increase of miR-21 levels in circulation of diabetic patients and in patients exhibiting dyslipidemia alone, compared to healthy individuals (Fig. 7A).

Similarly, people with diabetes or dyslipidemia revealed no significant modifications in circulating levels of miR-34a (Fig. 7B), one of the major miRNA involved in insulin production. MiR-34a was associated with insulin resistance being involved in pancreatic development, as well as in the onset of T_2_D [98, 102, 103]. There are contradictory data regarding circulating levels of miR-34a in diabetes. Plasma miR-34a was found reduced and negatively correlated with insulin and HOMA-IR [104]. Other data described increased expression of miR-34a in serum samples from T_2_D patients compared with pre-diabetic or T_2_D susceptible individuals [105].

Even if, we found no statistically modified expression of circulating miR-132 in diabetes or dyslipidemia (Fig. 7C), very recently, it was demonstrated that circulating levels of miR-132 were decreased in diabetic retinopathy patients in comparison to the ones without retinopathy, and they were inversely correlated with VEGF levels and oxidative stress markers [106].

Contradictory data also exist regarding mir-143 and miR-223 from circulation. Peripheral blood levels of miR-143 have been found down-regulated in the of patients with gestational diabetes [78], whereas elevated levels of miR-143 gene expression were correlated with the degree of obesity and proposed as predictor of insulin resistance in people without diabetes [107]. In line with previous studies, we also described a slightly increased level of miR-143 (Fig. 7D) in sera from patients with dyslipidemia, but this was not statistically significant. Likewise, in our study the differences in miR-223 expression were not significant between groups of patients with T_2_D, dyslipidemia and control group (Fig. 7E). Low levels of circulating miR-223 were considered marker for T_2_D onset [108]. Also, hyperglycemia induced increased levels of miR-223, in patients with acute coronary syndrome or stabile angina [109].

**Fig. 8:**
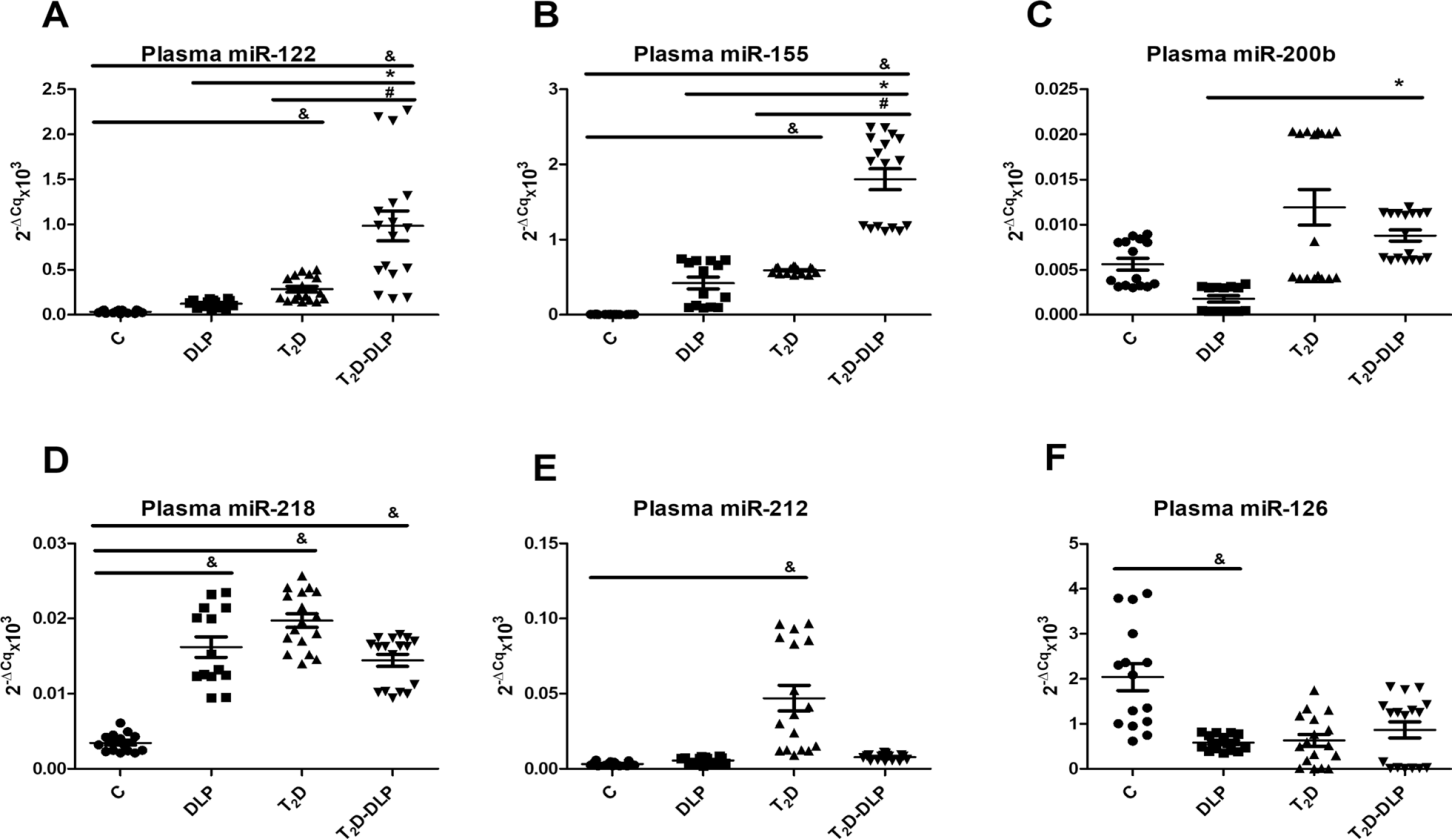
Relative expression (2^−ΔCq^ × 10^3^, ΔCq = Cq has-miRNA—Cq cel-miR-39) of miRNAs in plasma from patients with dyslipidemia (n = 14), diabetes (n = 17), diabetes associated with dyslipidemia (n = 17) and control subjects (n = 15): **A** miR-122-5p; **B** miR-155-5p; **C** miR 200-3p; **D** miR-218-5p; **E** miR-212-5p; **F** miR-126-3p. Data are expressed as means ± SD. ^&^*p* < 0.05 (vs C), ^*^p < 0.05 (vs DLP), ^#^ *p* < 0.05 (vs T_2_D) (One-Way ANOVA with Bonferroni's Multiple Comparison Test)

In our study we also investigated the levels of miR-122, miR-155, miR-200b, miR-218, miR-212 and miR-126 in plasma from DLP, T_2_D, T_2_D-DLP patient groups and control group (Fig. 8A–F). For miR-122 and miR-155 the expression levels were significantly higher in diabetic dyslipidemia (T_2_D-DLP group) compared to all other studied groups. Additionally, we found markedly increased levels of miR-122 (Fig. 8A) and miR-155 (Fig. 8B) in diabetic patients (T_2_D group) compared with the healthy subjects (C group). Circulating miR-122 has been found significantly up-regulated in individuals with T_2_D. A positive correlation between miR-122 and insulin levels, HOMA2-IR index [110], markers of obesity associated inflammation (TNFα, IL-1Ra, procalcitonin) [111–115] were also detected. Moreover, miR-122 has been associated with increased liver enzymes, adiposity, inflammation, and insulin resistance and with a contrasting lipid profile (higher triglycerides and lower HDL) [83]. Regarding mir 155, its expression in diabetes is a little controversial.

Dysregulation of miR-155 expression was proven to predict the severity of diabetes as well as the development of diabetes nephropathy, neuropathy, and retinopathy. A number of recent studies reported low serum levels of miR-155 in patients with T_2_D compared to healthy individuals [116–118]; while other data certify increased miR-155 in plasma or serum from T_1_D [119] and diabetes complications like nephropathy [120, 121] and retinopathy [92, 122]. MiR-155 was considered to be crucial for β-cell fitness [92]. Preclinical studies suggested that decreased miR-155 expression could be the key player in the mechanisms that underline the altered pancreatic function in T_2_D patients [117]. An insufficient level of miR-155 reduced β-cell growth in response to hypercholesterolemia [123], and only an up-regulation could help β-cell adaptation to overnutrition [92]. Any interference in this process could markedly contribute in the transition from pre-diabetes to T_2_D [92]. Furthermore high-fat diet altered the phenotype of cells in the islets of Langerhans, thereby insulin-secreting β-cells being re-differentiated into glucagon-producing α-cells [124].

For miR-200b, miR-218, miR-212 and miR-126, the expression levels were not significantly higher in diabetic dyslipidemia (T_2_D-DLP group) compared to all other studied groups. Nevertheless, the results revealed the higher levels of miR-200b in patients exhibiting T_2_D and dyslipidemia when compared to patients with dyslipidemia alone (Fig. 8C). Also, in our study, markedly elevated levels of plasma miR-218 were identified in all groups of patients investigated (DLP, T_2_D, T_2_D-DLP) compared to control group (Fig. 8D). In diabetes the expressions of circulating miR-218 and miR-200b have not been well described so far. It is known that, in diabetic retinopathy, the levels of miR-218 were up-regulated [125], while the levels of miR-200b were down-regulated [126].

Another miRNA associated with glucose imbalance is miR-212. It has been identified at children susceptible to metabolic disorders since the age of 16-and 17 years, later, at the age of 18, being associated with the onset of pre-diabetes [127]. We found circulating miR-212 significantly up-regulated in T_2_D group compared to control one (Fig. 8E). Even if, circulating miR-212 has been identified as marker of atherosclerosis and has been associated with three of the standard cardiovascular risk markers, HbA1c, HDL cholesterol, and lipoprotein a [128], in our study the levels of circulating miR-212 were found little but not markedly raised in patients with dyslipidemia or diabetes associated with dyslipidemia compared to healthy individuals.

Decreased miR-126 has been previously detected in T_2_D patients [129–131], and in diabetic patients with retinopathy [130]. Liu and collaborators, described miR-126 as potential marker for treatment response, showing that serum miR-126 increased after 6 months of diet and physical exercises in subjects with impaired glucose tolerance, as well as in T_2_D patients, to the latter insulin treatment being added [131]. The results were in line with data obtained by Ramezani-Aliakbari and collaborators on animal model. They showed protective effect of trimetazidine (a medicine used to prevent angina attacks), on the altered plasmatic levels of miR-126 in diabetic rats [132]. Together with its target gene IRS-1, miR-126 may be potential molecular target for the prevention and treatment of diabetic retinopathy. Also, circulating miR-126 has been associated with obesity and T_2_D and could be used to diagnose T_2_D at early stages [130, 131]. In line with these findings, we have also found, decreased levels of sera miR-126 in all groups of patients, the only statistically significant modification registered in the group of patients with dyslipidemia alone (Fig. 8F).

We then investigated possible correlations between significantly modified plasma miRNAs and clinical (Table 8) or inflammatory parameters (Table 9) in diabetes dyslipidemia. Besides the positive correlation between miR-200b with hemoglobin levels in plasma (r = 0.96, p = 0.007), negative associations were also observed between both miR-122 and miR-143 with creatinine (r = 0.90, p = 0.03 for miR-122; r = 0.89, p = 0.04 for miR-143) and diastolic blood pressure (r = 0.93, p = 0.02 for miR-122; r = 0.94, p = 0.01 for miR-143), and between miR-132 with uric acid (r = 0.88, p = 0.05) and erythrocytes (r = 0.95, p = 0.01) plasma levels (Table 8). Moreover, Il-1β plasma levels were positively correlated with miR-223 (r = 0.91, p = 0.03) and miR-155 (r = 0.89, p = 0.04) levels and negatively with miR-21 (r = 0.88, p = 0.05) plasma levels (Table 9). Also, TNFα levels were positively correlated with miR-21 (r = 0.94, p = 0.01) and miR-34a (r = 0.96, p = 0.01) and negatively correlated with miR-223 (r = 0.89, p = 0.04) (Table 9).

**Table 8:** Significant correlations among circulating/plasma miRNAs and relevant clinical parameters in the diabetic dyslipidemia context

T_2_D-DLP group		miR-21	miR-34a	miR-132	miR-143	miR-223	miR-122	miR-126	miR-155	miR-200b	miR-212	miR-218
Clinical measurements ↓	n	17	17	17	17	17	17	17	17	17	17	17
TC	r	− 0.052	− 0.154	− 0.691	0.324	0.061	0.021	0.030	− 0.052	− 0.451	− 0.340	0.441
	P	0.931	0.803	0.192	0.592	0.921	0.973	0.961	0.933	0.441	0.572	0.463
HDL-c	r	0.293	0.462	**0.821**	− 0.201	− 0.254	0.074	0.051	− 0.132	0.112	0.574	− 0.171
	P	0.642	0.441	**0.080**	0.742	0.683	0.902	0.942	0.831	0.851	0.314	0.783
LDL-c	r	0.431	− 0.122	− 0.292	0.300	− 0.512	− 0.131	***0.902***	− 0.711	− 0.233	0.411	0.612
	P	0.461	0.840	0.630	0.613	0.381	0.842	***0.031***	0.170	0.704	0.490	0.270
TG	r	− 0.511	− 0.55	− 0.592	− 0.312	0.442	− 0.381	− 0.494	0.451	0.432	− ***0.931***	− 0.451
	P	0.372	0.332	0.291	0.600	0.452	0.523	0.394	0.443	0.492	***0.023***	0.442
TGO	r	− 0.492	− 0.521	− 0.380	− 0.552	0.404	− 0.540	− 0.562	0.472	0.631	− ***0.945***	− 0.680
	P	0.401	0.360	0.522	0.330	0.502	0.342	0.323	0.421	0.252	***0.012***	0.212
TGP	r	0.402	− 0.153	0.090	− 0.112	− 0.514	− 0.380	0.781	− 0.632	0.264	0.391	0.140
	P	0.501	0.810	0.881	0.850	0.374	0.531	0.123	0.251	0.670	0.512	0.821
Fasting glucose	r	0.430	0.131	0.540	− 0.781	− 0.542	− **0.840**	0.171	− 0.444	0.551	0.040	− 0.410
	P	0.473	0.832	0.343	0.120	0.354	**0.071**	0.774	0.462	0.342	0.943	0.493
HbA1c	r	− 0.311	− 0.540	− 0.500	0.802	0.312	0.720	0.612	0.061	− 0.050	0.381	0.380
	P	0.600	0.341	0.363	0.100	0.614	0.170	0.271	0.920	0.931	0.520	0.524
Haemoglobin	r	− 0.423	− 0.460	0.070	− 0.753	0.311	− 0.584	− 0.462	0.423	***0.962***	− 0.652	− ***0.940***
	P	0.481	0.432	0.904	0.140	0.614	0.302	0.431	0.471	***0.004***	0.230	***0.012***
Creatinine	r	− 0.010	− 0.170	0.170	− ***0.893***	− 0.101	− ***0.900***	− 0.312	0.030	0.733	− 0.594	− 0.710
	P	0.984	0.783	0.784	***0.040***	0.874	***0.034***	0.604	0.961	0.161	0.292	0.173
Urea	r	− 0.180	− 0.370	− 0.340	− 0.5631	0.084	− 0.712	− 0.303	0.112	0.464	− 0.781	− 0.440
	P	0.772	0.541	0.583	0.320	0.903	0.174	0.620	0.850	0.433	0.110	0.451
Uric acid	r	0.620	− 0.580	− ***0.880***	0.293	0.621	0.202	− 0.340	0.531	− 0.21	− 0.713	− 0.020
	P	0.261	0.304	***0.054***	0.633	0.263	0.741	0.572	0.360	0.972	0.172	0.962
Erythrocytes	r	0.521	− 0.591	− ***0.950***	0.493	0.512	0.302	− 0.016	0.350	− 0.154	− 0.511	0.210
	P	0.372	0.290	***0.010***	0.401	0.380	0.621	0.920	0.562	0.802	0.382	0.734
Platelets	r	0.850	0.701	0.390	0.340	− **0.800**	0.123	0.712	− **0.860**	− 0.780	***0.910***	**0.844**
	P	0.063	0.184	0.514	0.571	**0.100**	0.851	0.180	**0.061**	0.121	***0.030***	**0.072**
Leucocytes	r	0.371	0.084	0.563	− 0.370	− 0.451	− 0.364	0.463	− 0.432	0.450	0.451	− 0.200
	P	0.543	0.901	0.324	0.534	0.440	0.541	0.430	0.463	0.453	0.440	0.743
Monocytes	r	− 0.190	0.342	0.013	0.260	0.334	0.474	− 0.594	0.422	− 0.502	− 0.132	0.122
	P	0.754	0.574	0.992	0.674	0.593	0.423	0.292	0.473	0.381	0.840	0.851
SBP	r	− 0.831	− 0.752	− 0.514	0.510	**0.830**	0.714	− 0.061	0.701	0.370	− 0.153	− 0.233
	P	0.084	0.140	0.372	0.384	**0.083**	0.182	0.922	0.192	0.543	0.804	0.711
DBP	r	0.064	− 0.053	0.262	− ***0.942***	− 0.174	− ***0.931***	− 0.362	− 0.011	0.662	− 0.554	− 0.702
	P	0.921	0.932	0.664	***0.014***	0.782	***0.020***	0.553	0.980	0.221	0.333	0.191

Spearman rank correlation test was used to assess relationships among variables. Bolded coefficients show a powerful correlation, and of them only italic underlined coefficients are significant (p<0.05)

**Table 9:** Significant correlations among circulating/plasma inflammatory cytokines and miRNAs in the diabetic dyslipidemia context

T_2_D-DLP group		IFNγ	TNFα	IL-1β	IL-6
Plasma miRNAs ↓	n	17	17	17	17
Plasma miR-21	r	0.251	***0.941***	− ***0.884***	− 0.352
	P	0.682	***0.013***	***0.052***	0.563
Plasma miR-34a	r	− 0.111	***0.962***	− 0.580	− 0.711
	P	0.854	***0.010***	0.302	0.174
Plasma miR-132	r	0.252	0.781	− 0.311	− 0.332
	P	0.680	0.122	0.612	0.584
Plasma miR-143	r	0.091	− 0.200	0.283	− 0.412
	P	0.880	0.751	0.651	0.480
Plasma miR-223	r	− 0.312	− ***0.894***	***0.913***	0.201
	P	0.600	***0.042***	***0.034***	0.742
Plasma miR-122	r	0.033	− 0.280	0.623	− 0.504
	P	0.962	0.643	0.262	0.383
Plasma miR-126	r	0.721	0.282	− 0.453	0.042
	P	0.160	0.641	0.441	0.951
Plasma miR-155	r	− 0.421	− 0.784	***0.892***	0.152
	P	0.421	0.124	***0.040***	0.801
Plasma miR-200b	r	0.252	− 0.572	0.412	**0.822**
	P	0.671	0.314	0.491	**0.083**
Plasma miR-212	r	0.620	0.681	− 0.322	− 0.532
	P	0.262	0.210	0.594	0.363
Plasma miR-218	r	0.100	0.452	− 0.462	− 0.564
	P	0.871	0.441	0.431	0.322

Spearman rank correlation test was used to assess relationships among variables. Bolded coefficients show a powerful correlation, and of them only italic underlined coefficients are significant (p < 0.05)

Association of plasma miRNAs with circulating MVs and some of their subtypes in plasma of diabetes patients with dyslipidemia (Table 10), revealed positive powerful correlations between miR-126 and PMVs (r = 0.86, p = 0.05) and miR-155 and total MVs (r = 0.97, p = 0.005), and negative correlations between miR-218 and ErMVs (r = 0.94, p = 0.01) (Table 10).

**Table 10:** Significant correlations among circulating/plasma MVs and miRNAs in the diabetic dyslipidemia context

T_2_D-DLP group		ErMVs	PMVs	LeMVs	MMVs	EMVs	total MVs
Plasma miRNAs ↓	n	17	17	17	17	17	17
Plasma miR-21	r	0.461	− 0.011	0.101	− 0.434	0.263	0.602
	P	0.425	0.982	0.872	0.462	0.672	0.271
Plasma miR-34a	r	− 0.022	0.291	− 0.264	− 0.050	0.154	− 0.820
	P	0.971	0.633	0.664	0.921	0.793	0.083
Plasma miR-132	r	− 0.792	− 0.401	− 0.274	0.580	− 0.490	− 0.601
	P	0.101	0.490	0.642	0.292	0.394	0.270
Plasma miR-143	r	− 0.750	− 0.614	0.223	− 0.191	− 0.650	− 0.472
	P	0.141	0.262	0.712	0.750	0.223	0.410
Plasma miR-223	r	− 0.162	− 0.114	− 0.590	0.603	0.054	− 0.134
	P	0.780	0.852	0.281	0.281	0.930	0.830
Plasma miR-122	r	− 0.401	− 0.480	0.672	− 0.682	− 0.552	− 0.044
	P	0.503	0.403	0.210	0.203	0.331	0.940
Plasma miR-126	r	0.754	***0.860***	− 0.331	− 0.122	0.760	− 0.392
	P	0.162	***0.051***	0.580	0.840	0.132	0.504
Plasma miR-155	r	0.174	− 0.282	0.692	− 0.513	− 0.181	***0.972***
	P	0.782	0.630	0.190	0.370	0.753	***0.005***
Plasma miR-200b	r	0.393	0.751	− 0.752	0.311	0.674	− 0.843
	P	0.501	0.133	0.134	0.600	0.212	0.074
Plasma miR-212	r	− 0.352	− 0.783	0.741	− 0.712	− 0.621	0.553
	P	0.550	0.111	0.142	0.170	0.260	0.334
Plasma miR-218	r	**− *0.942***	− 0.630	0.121	0.394	− 0.773	− 0.331
	P	***0.011***	0.244	0.843	0.514	0.121	0.570

Spearman rank correlation test was used to assess relationships among variables. Bolded coefficients show a powerful correlation, and of them only italic underlined coefficients are significant (p < 0.05)

Finally, we followed the relationships between plasma miRNAs and microvesicle-associated miRNAs (MV miRNAs) (Table 11) and found powerful positive correlations between the levels of plasma miR-21 and MV miR-223 (r = 0.95, p = 0.01), plasma miR-132 and MV miR-132 (r = 0.88, p = 0.04) and plasma miR-122 and MV miR-126 (r = 0.90, p = 0.03) (Table 11). In addition, some significant negative correlations between plasma miR-223 and MV miR-223 (r = 0.98, p = 0.002), plasma miR-126 and MV miR-122, (r = 0.90, p = 0.03) and plasma miR-155 and MV miR-223 (r = 0.97, p = 0.003) were also identified (Table 11).

**Table 11:** Significant correlations among MV miRNAs and plasma miRNAs in the diabetic dyslipidemia context

T_2_D-DLP group		MV miR-21	MV miR-34a	MV miR-132	MV miR-143	MV miR-223	MV miR-122	MV miR-126	MV miR-155	MV miR-200b	MV miR-212	MV miR-218
Plasma miRNAs ↓	n	17	17	17	17	17	17	17	17	17	17	17
Plasma miR-21	r	0.301	− 0.314	0.412	0.022	***0.951***	− 0.531	− 0.521	− 0.221	− 0.031	0.014	0.142
	P	0.612	0.614	0.481	0.961	***0.012***	0.350	0.361	0.711	0.952	0.972	0.812
Plasma miR-34a	r	0.263	− 0.032	0.410	0.462	0.610	0.022	− 0.510	− 0.204	− 0.112	0.391	0.251
	P	0.661	0.951	0.483	0.420	0.263	0.970	0.371	0.731	0.851	0.504	0.683
Plasma miR-132	r	− 0.361	0.274	***0.880***	0.761	0.490	0.1630	− 0.501	− 0.583	0.063	0.114	0.761
	P	0.542	0.652	***0.042***	0.130	0.391	0.781	0.384	0.304	0.912	0.852	0.122
Plasma miR-143	r	0.310	0.271	− 0.642	− 0.5023	− 0.230	− 0.220	**0.832**	− 0.061	0.591	− 0.383	− **0.843**
	P	0.601	0.652	0.231	0.380	0.702	0.714	**0.073**	0.914	0.282	0.522	**0.071**
Plasma miR-223	r	− 0.260	0.373	− 0.440	0.031	− ***0.980***	0.600	0.561	0.212	0.051	0.043	− 0.192
	P	0.663	0.530	0.443	0.950	***0.002***	0.272	0.321	0.721	0.920	0.941	0.751
Plasma miR-122	r	0.011	0.621	− 0.470	− 0.103	− 04.52	0.190	***0.901***	− 0.233	0.664	− 0.283	− 0.582
	P	0.980	0.260	0.412	0.860	0.035	0.744	***0.032***	0.704	0.212	0.641	0.291
Plasma miR-126	r	0.122	− 0.083	0.010	− 0.603	0.612	− ***0.904***	0.281	− 0.381	0.560	− 0.754	− 0.342
	P	0.840	0.890	0.971	0.280	0.250	***0.034***	0.640	0.521	0.323	0.140	0.561
Plasma miR-155	r	− 0.321	0.381	− 0.280	0.274	− ***0.97***	0.782	0.362	0.213	− 0.082	0.230	0.025
	P	0.581	0.510	0.644	0.650	***0.003***	0.110	0.540	0.730	0.891	0.700	0.960
Plasma miR-200b	r	− 0.744	0.142	0.392	0.214	− 0.351	0.232	− 0.071	− 0.122	− 0.083	− 0.211	0.611
	P	0.141	0.814	0.501	0.720	0.554	0.701	0.902	0.840	0.894	0.724	0.263
Plasma miR-212	r	− 0.022	0.344	0.324	0.014	0.592	− 0.464	0.243	− 0.701	0.692	− 0.502	− 0.071
	P	0.972	0.573	0.591	0.980	0.290	0.422	0.691	0.182	0.193	0.381	0.904
Plasma miR-218	r	0.651	− 0.173	− 0.404	− 0.522	0.471	− 0.593	0.254	− 0.014	0.311	− 0.183	− 0.712
	P	0.224	0.770	0.493	0.360	0.414	0.281	0.672	0.981	0.614	0.761	0.170

Spearman rank correlation test was used to assess relat1ionships among variables. Bolded coefficients show a powerful correlation, and of them only italic underlined coefficients are significant (p < 0.05)

Regarding the analysis of miRNAs in plasma, we can conclude that the levels of miR-21, miR-34a, miR-132, miR-143, miR-223, miR-122, miR-155, miR-200b and miR-218 were up-regulated in diabetic dyslipidemia (T_2_D-DLP group) as a cumulative result of the alterations induced by both T_2_D and dyslipidemia. These plasma miRNAs found modified in diabetic dyslipidemia presented significant correlations with some important markers associated with this pathology. Thus: plasma miR-21 was positively correlated with TNFα plasma levels and MV miR-223 and negatively correlated with Il-1β plasma levels; plasma miR-34a was positively associated with TNFα plasma levels; plasma miR-132 was positively connected with MV miR-132 and negatively with uric acid and erythrocytes plasma levels; plasma miR-143 was negatively related with creatinine and diastolic blood pressure; plasma miR-223 was positively linked with Il-1β plasma levels and negatively with TNFα plasma levels and MV miR-223 levels; plasma miR-122 was positively associated with MV miR-126 levels and negatively with creatinine and diastolic blood pressure; plasma miR-155 was positively correlated with Il-1β plasma levels and total MV levels and negatively with MV miR-223 levels; plasma miR-200b was positively linked with hemoglobin levels and plasma miR-218 was negatively related with the levels of ErMVs.

Of all, in diabetes dyslipidemia, alterations of two of the above miRNAs, were related with one of the diseases more than with the other one. Thus, the modified miR-122 was induced by dyslipidemia more than by diabetes in both plasma MVs and the whole circulation, whereas for miR-132 the association was obvious especially within plasma MVs. Because neither in diabetes, nor in dyslipidemia, the levels of miR-21, miR-143, miR-155 and miR-218 were not significantly modified, we can assume that the changes in their expression were the result of imbalances occurred in diabetes dyslipidemia itself more than in diabetes or dyslipidemia alone.

Taking into account that miRNAs contained in MVs significantly up-regulated were miR-218, miR-212, miR-200b and miR-143, and those significantly down-regulated were miR-21, miR-122, miR-126 and miR-155, and miRNAs in plasma significantly up-regulated were miR-21, miR-34a, miR-132, miR-143, miR-223, miR-122, miR-155 and miR-218, we can conclude that miRNAs common to MVs and plasma significantly increased were miR-218, miR-132 and miR-143. In addition, three miRNAs common to MV and plasma, namely miR-21, miR-122 and miR-155, which evolved in different directions of decrease and increase, respectively, were identified.

**Fig. 9:**
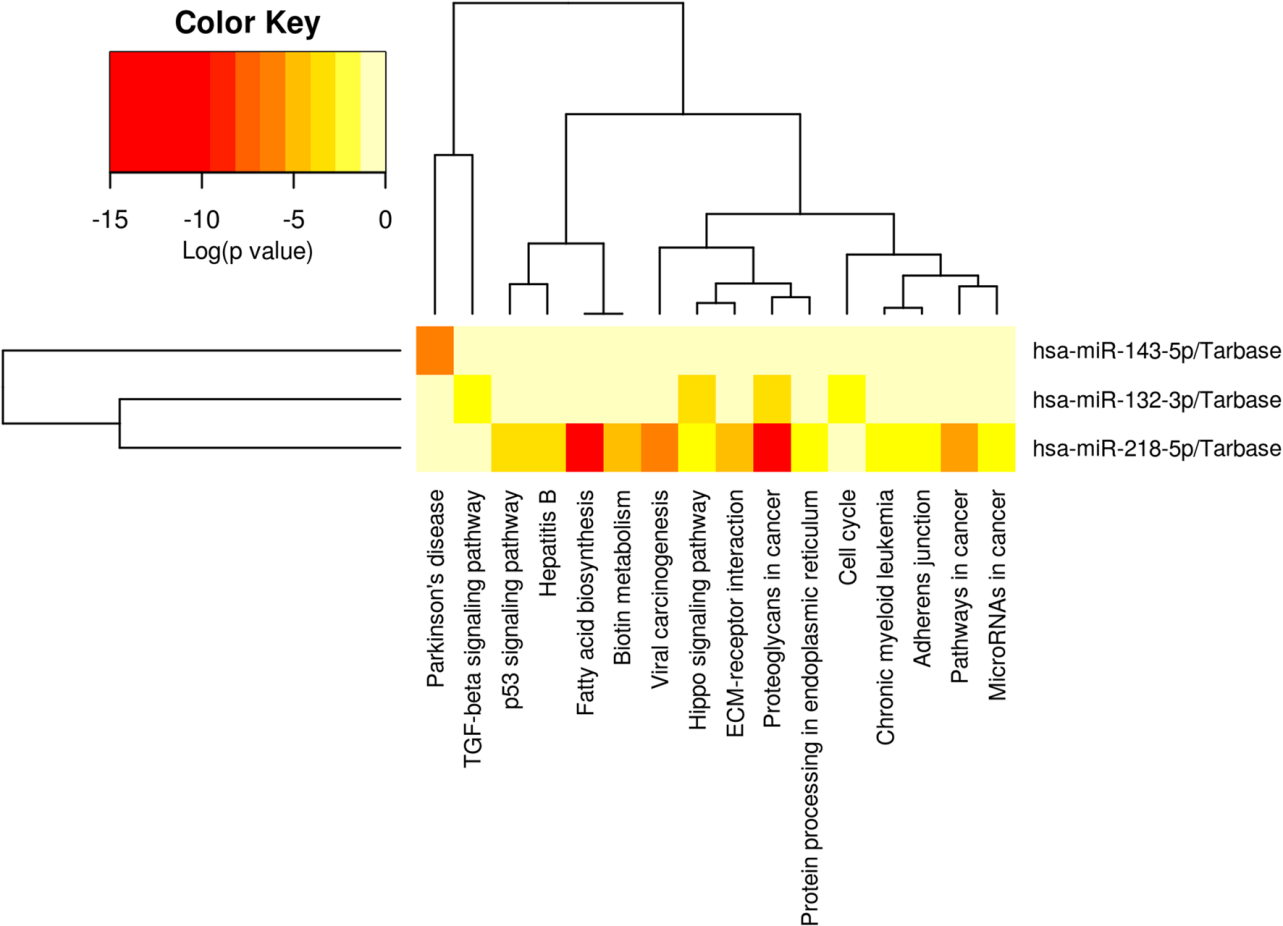
Heat map showing three similarly expressed miRNAs from circulation and MVs in T_2_D-DLP patients (n = 17)

It is interesting to report that in diabetic dyslipidemia, of the three miRNAs whose levels were found higher in circulation as well as within plasma MVs, miR-143 was associated with Parkinson’s disease, miR-132 with hippo signaling pathway and proteoglycans in cancer, and miR-218 with genes involved in fatty acids biosynthesis, proteoglycans in cancer, viral carcinogenesis, pathways in cancer, biotin metabolism, ECM-extracellular matrix receptor interaction, hepatitis B, p53 signaling pathway (Fig. 9).

**Fig. 10:**
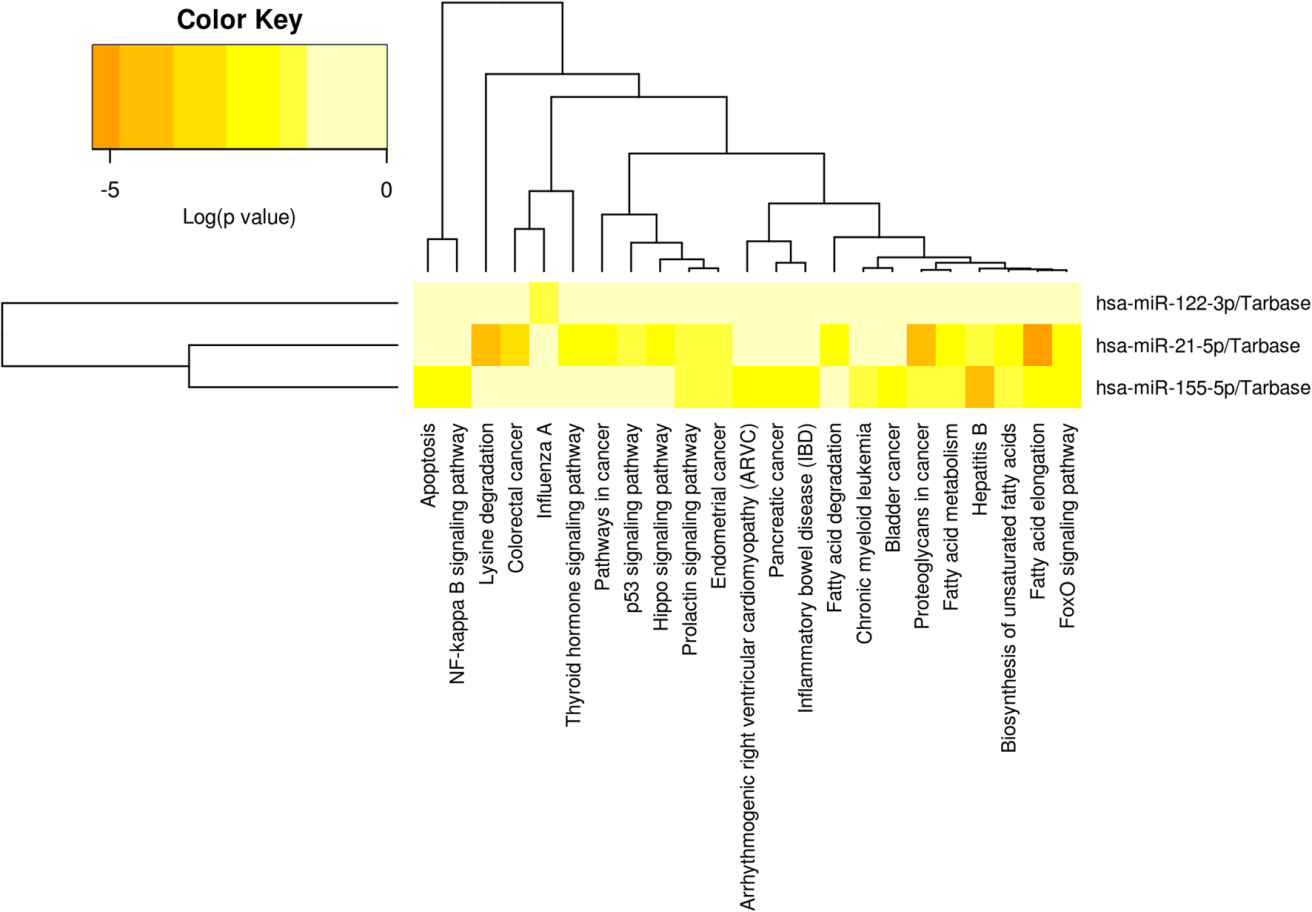
Heat map showing three differentially expressed miRNAs from circulation and MVs in T_2_D-DLP patients (n = 17)

Also, two of the three miRNAs found to be expressed differently in plasma and MVs of diabetic dyslipidemic patients, respectively miR-21 and miR-155, were associated with genes involved in various pathologies, namely: miR-21 in lysine degradation, fatty acid degradation, proteoglycans in cancer, colorectal cancer, and miR-155 in hepatitis B (Fig. 10).

## Strengths and limitations of the study

The study has several strong points that deserve to be considered**: **(1) The patients enrolled in the study did not have obesity or other disorders or even cardiovascular diseases. Also, they have not been exposed to infection or vaccination with COVID-19. Any of these conditions could have been potential sources of variation and could have affected the relevance of the study in the context of the investigated pathology, diabetic dyslipidemia; (2) The results regarding the expression of miRNAs in microvesicles and plasma were correlated with several metabolic and inflammatory parameters, constituting the basis for the development of future therapeutic strategies; (3) Identification of a specific set of miRNAs (miR-218, miR-132, miR-143, and miR-21, miR-122, miR-155), common to microvesicles and plasma and with a modified expression profile in diabetic dyslipidemia, makes these miRNAs to be considered potential biomarkers for the diagnosis and prognosis of this pathology, but also specific targets for the application of an effective therapy.

There are limitations of the study that need to be mentioned: (1) MVs collected from plasma isolated from all the participants, were stored at − 80 °C until use but not more than 2–3 weeks. Still, all the tests performed for their characterization proved that their number and structure were not affected by the freezing/thawing procedure; (2) Experiments characterizing MVs (large EVs) by Zetasizer Nano ZS, fluorescence and electron microscopy showed the presence of an extremely small, even negligible, percentage of smaller vesicles (exosomes/small EVs). It is important to note that, the majority population of vesicles was found in a range of values specific to MVs (100–1000 nm); (3) Circulating miRNAs was not quantified in the plasma depleted of MVs to be able to state with certainty that the miRNAs detected in the plasma belong to a rather large percentage to the MVs that the plasma contains; (4) To understand the origin of miRNAs expressed differently in MVs, the sorting of different types of MVs may help in future experiments; (5) Most of the patients with diabetes dyslipidemia were under treatment for diabetes and dyslipidemia and their BMI was higher compared to that of diabetic patients and control subjects; (6) Because these data are cross-sectional, a concluding remark on miRNA and MV evolution characteristic to a particular patient cannot be drown.

Despite all these limitations, the strengths of this study make our data pathophysiological relevant, demonstrating that MV levels, as well as their miRNA content, can represent a consistent tool for the early detection of diabetes complications.

Nevertheless, additional prospective studies are needed to certify the actual relevance of miR-218, miR-132, miR-143, and miR-21, miR-122, miR-155 as diagnostic and prognostic biomarkers of diabetes dyslipidemia in the overall population.

## Conclusions

Our findings suggest that the presence of dyslipidemia more than diabetes in the pathology of diabetes dyslipidemia, seems to play an important role in the up-regulation and down-regulation of some miRNAs, such as miR-218, miR-132, miR-143, and miR-21, miR-122, miR-155, detected in both plasma and circulating MVs.

Up-regulated miR-218, miR-132 and miR-143 and down-regulated miR-21, miR-122, and miR-155 were associated with important plasma markers representative of diabetes dyslipidemia, but also with different signaling pathways, including p53 signaling pathway and hippo signaling pathway, and other pathologies such as Parkinson’s disease, colorectal cancer, hepatitis B. Also, these miRNAs were shown to control some process within the body: miR-218, miR-132 and miR-143 to control fatty acids biosynthesis, biotin metabolism, extracellular matrix-receptor interaction, and miR-21, miR-122, and miR-155 lysine and fatty acid degradation.

Understanding the regulatory networks of miR-143-3p, miR-132-3p, miR-218-5p and miR-21, miR-122, and miR-155 is essential for the development of therapies for diabetes dyslipidemia as well as for the identification of new biomarkers which will reflect the severity as well as the progression of the disease.

## Data Availability

The original contributions presented in the study are included in the article, additional inquiries can be addressed to the corresponding author.

## Abbreviations

miRNAs: MicroRNAs
MVs: Microvesicles
DDLP: Diabetic dyslipidemia
LDL: Low-density lipoprotein
HDL: High-density lipoprotein
T_2_D: Type 2 diabetes
PS: Phosphatidylserine
EMPs: Endothelial microparticles
PMPs: Platelet microparticles
LeMPs: Leukocyte microparticles
ECs: Endothelial cells
EPCs: Endothelial progenitor cells
mRNA: Messenger RNA
BMI: Body mass index
T_2_D-DLP: Type 2 diabetes and dyslipidemia
DLP: Dyslipidemia
C: Control
HbA1C: Glycated haemoglobin
IL-6: Interleukin 6
IL-1β: Interleukin 1beta
IFNγ: Interferon gamma
TNFα: Tumor necrosis factor alfa
PFP: Phosphate buffered saline
FBS: Fetal bovine serum
RT: Room temperature
qRT-PCR: Quantitative reverse transcription polymerase chain reaction
snRNA: Small nuclear RNA
Cq: Quantification cycle
EMVs: Microvesicles derived from endothelial cells
PMVs: Microvesicles derived from platelets
LeMVs: Microvesicles derived from leucocytes
MMVs: Microvesicles derived monocytes
ErMVs: Microvesicles derived from erythrocytes
PreDM: Pre-diabetic
NGT: Normal glucose tolerance
MV miRNAs: Microvesicle-associated microRNAs
